# The L-proline modified Zr-based MOF (Basu-proline) catalyst for the one‐pot synthesis of dihydropyrano[3,2-*c*]chromenes

**DOI:** 10.1038/s41598-023-44774-4

**Published:** 2023-10-17

**Authors:** Amin Benrashid, Davood Habibi, Masoumeh Beiranvand, Maryam Mahmoudiani Gilan

**Affiliations:** Department of Organic Chemistry, Faculty of Chemistry, Bu-Ali Sina University, Hamedan, Iran

**Keywords:** Chemistry, Materials science

## Abstract

A novel, reusable, and efficient L-proline-modified Zr-based metal–organic framework (Basu-proline) was designed, synthesized, and characterized by Fourier Transform-Infrared spectroscopy (FT-IR), Energy-Dispersive X-ray spectroscopy (EDX), elemental mapping, Field Emission Scanning Electron Microscopy (FE-SEM), X-ray Diffraction analysis (XRD), Thermo-Gravimetric-Differential Thermal Analysis (TGA-DTA)*,* and N_2_ adsorption–desorption isotherms. Then, its catalytic performance was assessed in the synthesis of dihydropyrano[3,2-*c*]chromenes via the one-pot, three‐component tandem condensation reaction of 4-hydroxycoumarin, aromatic aldehydes and malononitrile. The Basu-proline catalyst exhibited a better efficiency than some reported protocols regarding higher yields, lower reaction times, and simple separation.

## Introduction

Metal–organic frameworks (MOFs) are an emerging type of porous coordination network with promising catalytic capabilities that fabricate by self-assembly from a diversity of organic ligands and inorganic metal clusters^1^. MOFs can be designed for specific purposes by choosing the appropriate and favored organic ligands, functional groups, and metal centers that preferably attach to specific molecules^2^. Due to their large porosity, rich coordination chemistry, and synthetic tunability, they have found wide applications in gas adsorption/separation, drug delivery, heterogeneous catalysis, chemical sensing, microelectronics, and water purification^3–6^. Zr-based MOFs demonstrate high water/moisture, chemical, and thermal stability due to the strong Zr-O bonds between the Zr(IV) cations and the carboxylate ligands as hard acid–base, respectively. They are also stable in acidic and some basic solutions^7–10^.

One of the privileged chiral catalysts is L-proline and its derivatives used as organocatalysts to catalyze reactions such as Mannich, Michael, Morita–Baylis–Hillman reactions, and the aldol addition^11^. Due to the high porosity of MOFs, the proline can be anchored onto the MOF to produce the heterogeneous catalyst with high enantioselectivity^12^.

Chromenes are a group of heterocyclic biological compounds found in many natural products (flavonoids and alkaloids) and pharmaceutically useful intermediates^13,14^. Dihydropyrano [3,2-*c*]chromenes have shown potential application in treating diseases such as Alzheimer, Parkinson, Schizophrenia, Myoclonus, Huntington, and Down’s syndrome^15^. So far, several synthetic methods have been reported for preparation of dihydro-pyrano[3,2-*c*]chromenes with different catalysts such as PNO-Ag_2_O^16^, dehydroabietylamine/cinchonine/squaramide^17^, urea^18^, MNPs-PhSO_3_H^19^, Fe_3_O_4_@GO/naphthalene-SO_3_H^20^, DMAP^21^, ZHY@SiO_2_-Pr-Py^22^, Ni(II)/Schiff base/SBA-15^23^, H_5_BW_12_O_40_^24^, [γ-Fe_2_O_3_@HapSi(CH_2_)_3_ AMP]^25^, BPMO@ ISB/Mn(II)^26^, 2-hydroxyethylammonium formate^27^, tertiary amine-thiourea^28^, 2-hydroxyethanaminium formate, 3-hydroxypropanaminium formate, 2-hydroxyethanaminium acetate and 3-hydroxypropanaminium acetate^29^. Despite the presentation of various catalysts, the scope and selectivity of these methods should be promoted. These protocols have disadvantages such as low catalytic activity, harsh reaction conditions, lack of isolation and reusability, etc.

**Scheme 1:**
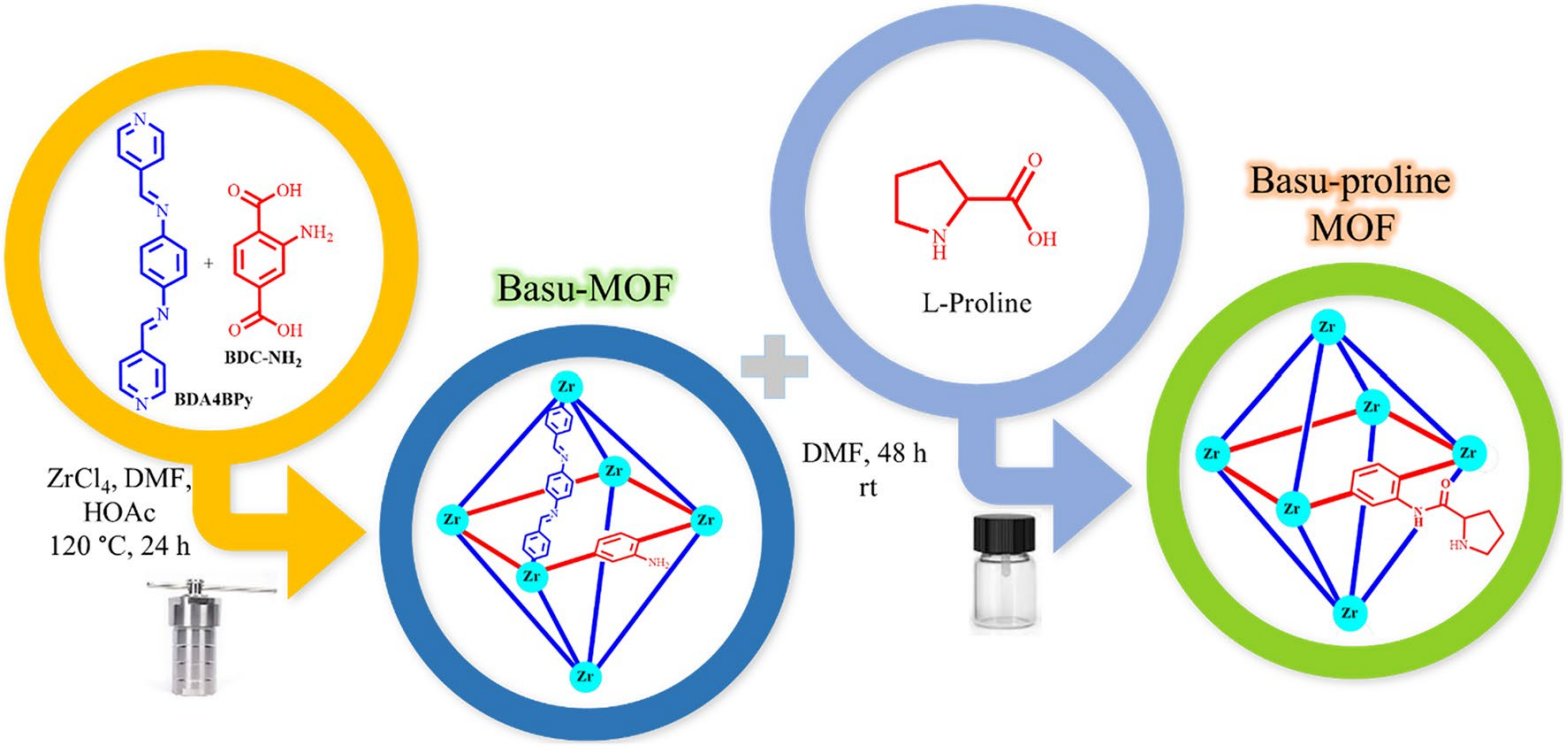
Synthesis of Basu-proline MOF.

Herein, we wish to report the preparation of Basu-proline and its characterization with various techniques such as FT-IR, EDX, elemental mapping, FE-SEM, TGA-DTA, XRD, and N_2_ adsorption–desorption isotherms (Scheme 1).

**Scheme 2:**
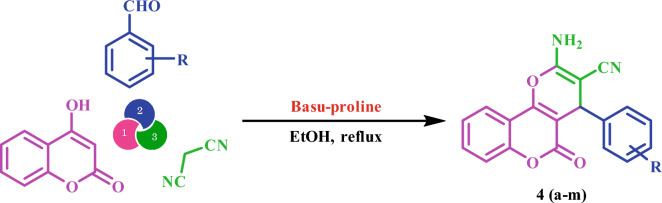
Synthesis of **4(a-m)**.

Then, Basu-proline was used as a capable catalyst to accelerate the synthesis of dihydro- pyrano[3,2-*c*]chromenes **4(a-m)** via the three-component condensation reaction of 4-hydroxy-coumarin, aromatic aldehydes, and malononitrile (Scheme 2). The advantages of presented catalyst include short reaction time, high efficiency, low catalyst loading, reusability of catalyst, recyclability, and compatibility with electron‐donating and electron‐withdrawing groups.

## Experimental section

### General

All chemicals were provided by the Merck and Aldrich chemical companies and used as received. The reaction progress and purity of the synthesized compounds were monitored by TLC (silica gel 60 F-254 plates). The FT‐IR spectra were taken on a Perkin Elmer instrument 10.02.00 employing KBr pellets. The ^1^H NMR (250 MHz) and ^13^C NMR (62.5 MHz) spectra were recorded on a Bruker spectrometer (δ in ppm) using DMSO‐*d*_*6*_ as a solvent. Melting points were measured with a BUCHI 510 melting point apparatus. The elemental analyses were performed using the MIRA II analyze, and the FE-SEM images were obtained using the MIRA III analyzer. The XRD measurements were performed using the XRD Philips PW1730. TGA-DTA analysis was obtained with the SDT-Q600 instrument.

### General strategy for the fabrication of the Basu-proline

Basu-proline was prepared in the following two successive steps:

*Step 1*: The Basu-MOF was prepared by the procedure reported before^30^. Briefly, ZrCl_4_ (1.2 mmol), BDA4BPy (*N*^1^,*N*^4^-bis(pyridin-4-ylmethylene)benzene-1,4-diamine) ligand (0.3 mmol), and 2-aminoterephthalic acid (BDC-NH_2_) (0.5 mmol) were dissolved in DMF (140 mL) and stirred for 15 min at room temperature. Next, acetic acid (20 mL) was added, and the mixture was placed in a Teflon reactor and put in an oven at 120 °C for 24 h. Then, the mixture was allowed to cool slowly to room temperature, centrifuged, and washed with DMF and ethanol.

*Step 2*: The Basu-proline was prepared via the reported procedure^31^. Briefly, Basu-MOF (29 mg) was dispersed in DMF (2.5 mL) for 10 min at room temperature. Then, L-proline (10 mg) was added to the mixture and stirred for 48 h. The resulting cream-colored precipitate was centrifuged, washed with DMF (2 × 10 mL) and CH_3_OH (2 × 10 mL), and oven dried at 80 °C.

### General procedure for the synthesis of **4(a-m)** by Basu-proline

The mixture of 4-hydroxycoumarin (1 mmol), malononitrile (1 mmol), aromatic aldehydes (1 mmol), and Basu-proline (20 mg) was refluxed in ethanol (5 mL) for an appropriate time. After the reaction (TLC: n-hexane/EtOAc) was completed, the resulting precipitate was dissolved in DMF (5 mL) and centrifuged to separate the catalyst. Then, water was added to the resulting mixture, and since DMF is completely miscible in water, the obtained mixture was filtered and the solid was washed with hot ethanol to give dihydropyrano[3,2-*c*]chromenes in high yields.

### Spectral data

**2-Amino-4-(3,4-dimethoxyphenyl)-5-oxo-4** ***H*** **,5** ***H*** **-pyrano[3,2-** ***c*** **]chromene-3-carbonitrile (4a)** 
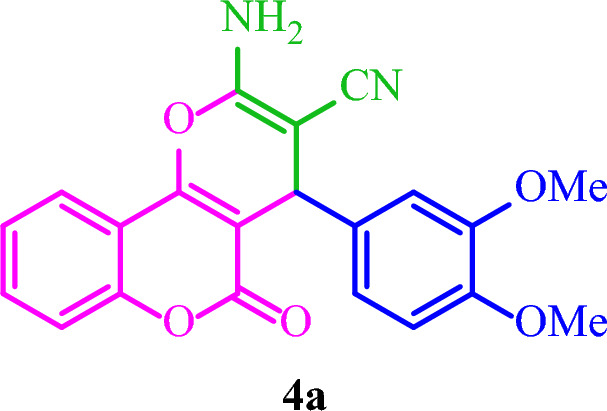


White solid; M.p.: 230–233 °C (reported M.p.: 217–219 °C^32^); IR (KBr): $\upsilon$ (cm^−1^) = 3406, 3326, 3261, 2196, 1709, 1673, 1378, 1048, 760. ^1^H NMR (250 MHz, DMSO-*d*_*6*_) δ = 7.87 (d, *J* = 7.8 Hz, 1H), 7.68 (t, *J* = 7.8 Hz, 1H), 7.45 (t, *J* = 9.3 Hz, 2H), 7.35 (s, 2H), 6.85 (d, *J* = 12.4 Hz, 2H), 6.72 (d, *J* = 8.3 Hz, 1H), 4.38 (s, 1H), 3.69 (s, 6H). ^13^C NMR (62.5 MHz, DMSO-*d*_*6*_) δ = 158.4, 152.5, 149, 149, 136.3, 133.3, 125.1, 122.9, 120.1, 119.8, 117, 112.3, 112, 104.5, 58.5, 55.9, 40.9, 40.6, 40.3, 39.9, 39.6, 39.3, 38.9, 36.9.

**2-Amino-5-oxo-4-phenyl-4** ***H*** **,5** ***H*** **-pyrano[3,2-** ***c*** **]chromene-3-carbonitrile (4b)** 
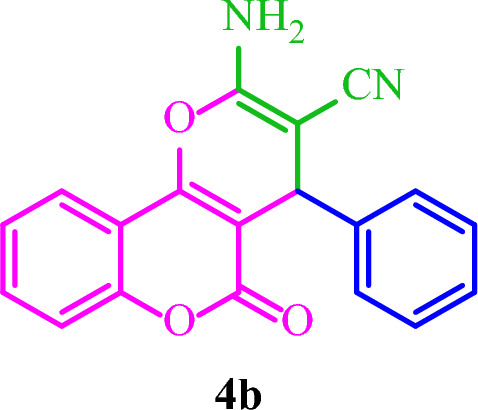


White solid; M.p.: 271–274 °C (reported M.p.: 271–273 °C^33^); IR (KBr): $\upsilon$ (cm^−1^) = 3379, 3285, 3181, 2199, 1709, 1675, 1383, 1059, 758. ^1^H NMR (250 MHz, DMSO-*d*_*6*_) δ = 7.89 (d, *J* = 8.0 Hz, 1H), 7.70 (t, *J* = 7.9 Hz, 1H), 7.45 (t, *J* = 6.6 Hz, 2H), 7.37 (s, 2H), 7.26 (dd, *J* = 13.2, 7.0 Hz, 5H), 4.43 (s, 1H). ^13^C NMR (62.5 MHz, DMSO-*d*_*6*_) δ = 158.4, 152.5, 143.7, 133.4, 128.9, 128, 127.5, 125.1, 122.9, 119.6, 117, 113.4, 104.4, 58.4, 37.4.

**2-Amino-4-(4-chlorophenyl)-5-oxo-4** ***H*** **,5** ***H*** **-pyrano[3,2-** ***c*** **]chromene-3-carbonitrile (4c)** 
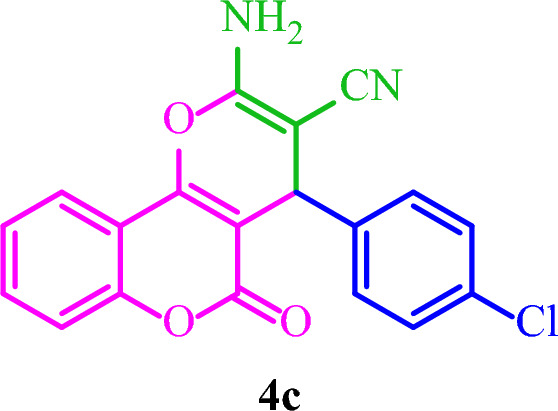


White solid; M.p.: 282–285 °C (reported M.p.: 270–271°C^33^); IR (KBr): $\upsilon$ (cm^−1^) = 3382, 3311, 3189, 2193, 1713, 1676, 1376, 1061, 759. ^1^H NMR (250 MHz, DMSO-*d*_*6*_) δ = 7.88 (d, *J* = 8.1 Hz, 1H), 7.68 (d, *J* = 7.7 Hz, 1H), 7.42 (s, 4H), 7.31 (d, *J* = 10.4 Hz, 4H), 4.46 (s, 1H). ^13^C NMR (62.5 MHz, DMSO-*d*_*6*_) δ = 159.9, 158.4, 154, 152.6, 142.7, 133.4, 132.1, 130, 128.8, 125.1, 122.9, 119.5, 117, 113.3, 103.9, 57.9, 36.8.

**2-Amino-4-(2-chlorophenyl)-5-oxo-4** ***H*** **,5** ***H*** **-pyrano[3,2-** ***c*** **]chromene-3-carbonitrile (4d)** 
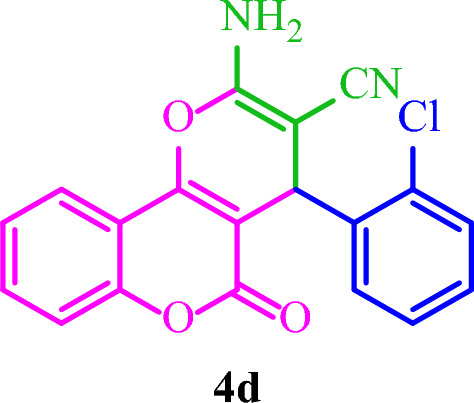


White solid; M.p.: 292–295 °C (reported M.p.: 270–273 °C^34^); IR (KBr): $\upsilon$ (cm^−1^) = 3402, 3285, 3180, 2201, 1709, 1675, 1380, 1063, 756. ^1^H NMR (250 MHz, DMSO-*d*_*6*_) δ = 7.88 (d, *J* = 7.8 Hz, 1H), 7.70 (t, *J* = 7.9 Hz, 1H), 7.43 (d, *J* = 12.9 Hz, 5H), 7.26 (s, 3H), 4.95 (s, 1H). ^13^C NMR (62.5 MHz, DMSO-*d*_*6*_) δ = 158.5, 152.6, 140.6, 133.4, 132.7, 131.1, 130, 129.2, 128.1, 125.1, 122.9, 119.1, 117.0, 113.2, 103.3, 56.9, 34.7.

**2-Amino-4-(2,4-dichlorophenyl)-5-oxo-4** ***H*** **,5** ***H*** **-pyrano[3,2-** ***c*** **]chromene-3-carbonitrile (4e)** 
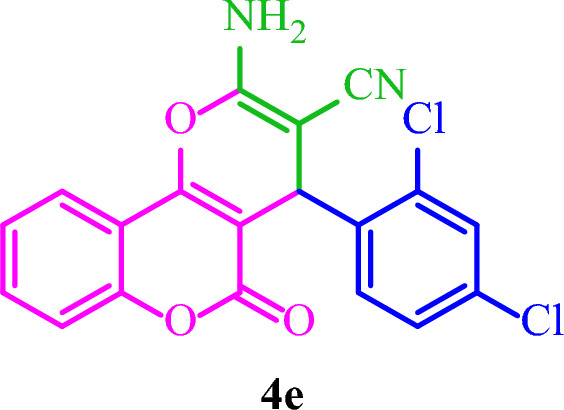


White solid; M.p.: 260–263 °C (reported M.p.: 258–260 °C^35^); IR (KBr): $\upsilon$ (cm^−1^) = 3463, 3297, 3164, 2200, 1716, 1674, 1376, 1062, 762. ^1^H NMR (250 MHz, DMSO) δ = 7.88 (d, *J* = 7.6 Hz, 1H), 7.70 (t, *J* = 7.6 Hz, 1H), 7.49 (q, *J* = 10.6 Hz, 5H), 7.35 (d, *J* = 3.1 Hz, 2H), 4.95 (s, 1H). ^13^C NMR (62.5 MHz, DMSO-*d*_*6*_) δ = 159.8, 158.5, 154.6, 152.6, 139.8, 133.8, 133.5, 132.8, 132.5, 129.2, 128.3, 125.1, 122.9, 119.1, 117, 113.2, 102.9, 56.4, 34.3.

**2-Amino-4-(3-nitrophenyl)-5-oxo-4** ***H*** **,5** ***H*** **-pyrano[3,2-** ***c*** **]chromene-3-carbonitrile (4f)** 
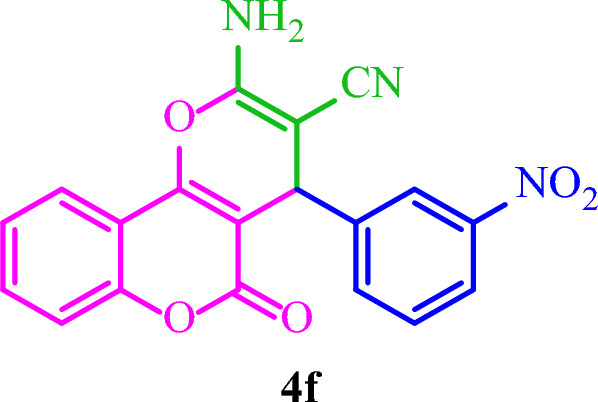


Yellow solid; M.p.: 262–265 °C (reported M.p.: 260–264 °C^35^); IR (KBr): $\upupsilon$ (cm^−1^) = 3408, 3324, 3195, 2193, 1711, 1677, 1379, 1064, 756. ^1^H NMR (250 MHz, DMSO-*d*_*6*_) δ = 8.09 (s, 2H), 7.94–7.22 (m, 8H), 4.69 (s, 1H). ^13^C NMR (62.5 MHz, DMSO-*d*_*6*_) δ = 160, 158.5, 154.3, 152.7, 148.3, 145.9, 135.1, 133.5, 130.4, 125.1, 122.9, 119.3, 117, 113.3, 103.3, 57.3, 40.6, 37.

**2-Amino-4-(4-nitrophenyl)-5-oxo-4** ***H*** **,5** ***H*** **-pyrano[3,2-** ***c*** **]chromene-3-carbonitrile (4g)** 
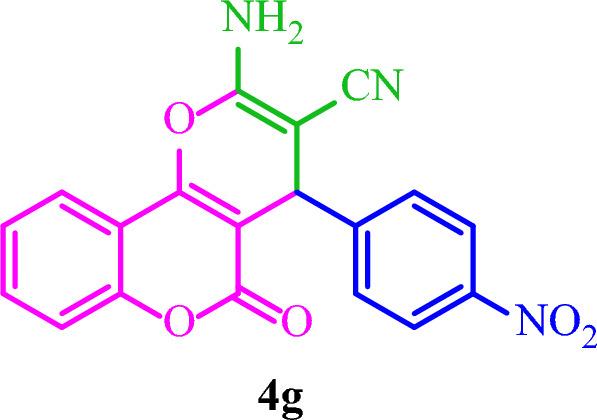


Yellow solid; M.p.: 285–288 °C (reported M.p.: 260–262 °C^36^); IR (KBr): $\upupsilon$ (cm^−1^) = 3482, 3430, 3369, 2195, 1717, 1671, 1376, 1061, 759.

**2-Amino-5-oxo-4-(** ***p*** **-tolyl)-4** ***H*** **,5** ***H*** **-pyrano[3,2-** ***c*** **]chromene-3-carbonitrile (4h)** 
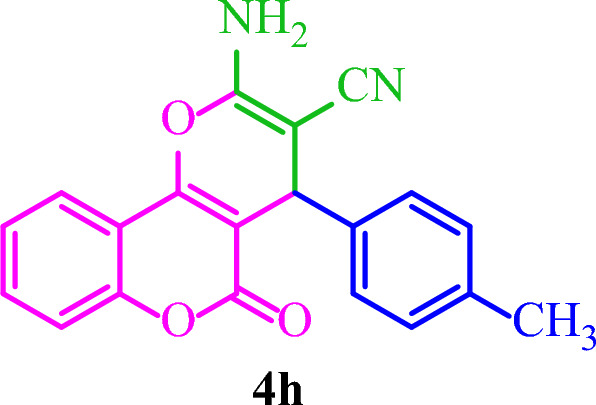


White solid; M.p.: 267–270 °C (reported M.p.: 262–265 °C^35^); IR (KBr): $\upsilon$ (cm^−1^) = 3390, 3311, 3261, 2195, 1713, 1678, 1382, 1058, 756.

**2-Amino-4-(4-isopropylphenyl)-5-oxo-4** ***H*** **,5** ***H*** **-pyrano[3,2-** ***c*** **]chromene-3-carbonitrile (4i)** 
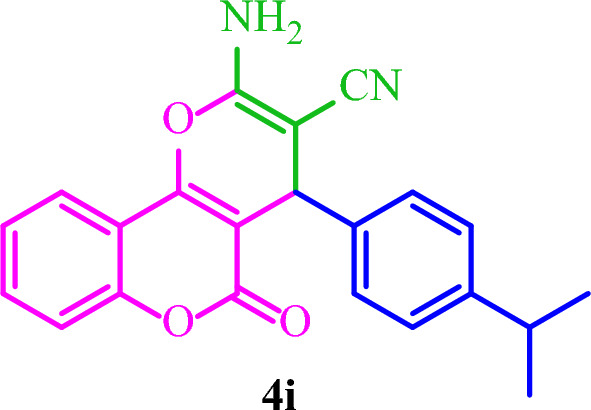


White solid; M.p.: 233–236 °C (reported M.p.: 239–241 °C^37^); IR (KBr): $\upsilon$ (cm^−1^) = 3390, 3304, 3205, 2202, 1713, 1672, 1375, 1050, 769.

**2-Amino-4-(3-hydroxyphenyl)-5-oxo-4** ***H*** **,5** ***H*** **-pyrano[3,2-** ***c*** **]chromene-3-carbonitrile (4j)** 
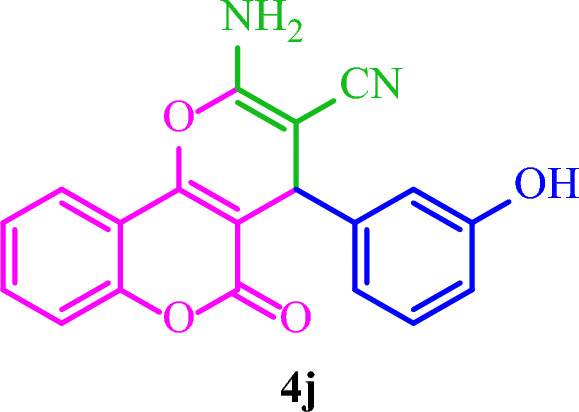


White solid; M.p.: 270–273 °C (reported M.p.: 258–260 °C^36^); IR (KBr): $\upsilon$ (cm^−1^) = 3442, 3332, 3182, 2203, 1721, 1676, 1376, 1058, 765.

**2-Amino-4-(2,3-dihydroxyphenyl)-5-oxo-4** ***H*** **,5** ***H*** **-pyrano[3,2-** ***c*** **]chromene-3-carbonitrile (4k)** 
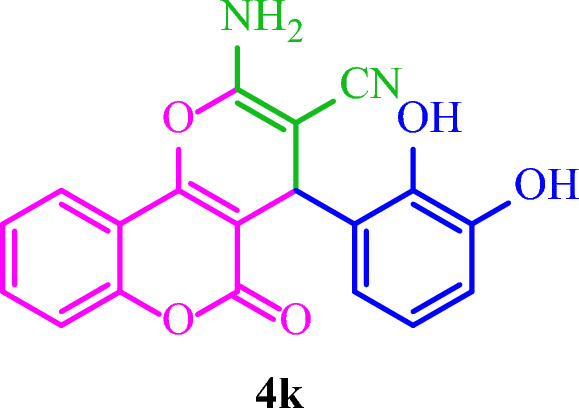


Yellow solid; M.p.: 249–252 °C (reported M.p.: 258 °C^38^); IR (KBr): $\upsilon$ (cm^−1^) = 3348, 3185, 3100, 2203, 1678, 1612, 1385, 1082, 755.

**2-Amino-4-(2-hydroxyphenyl)-5-oxo-4** ***H*** **,5** ***H*** **-pyrano[3,2-** ***c*** **]chromene-3-carbonitrile (4l)** 
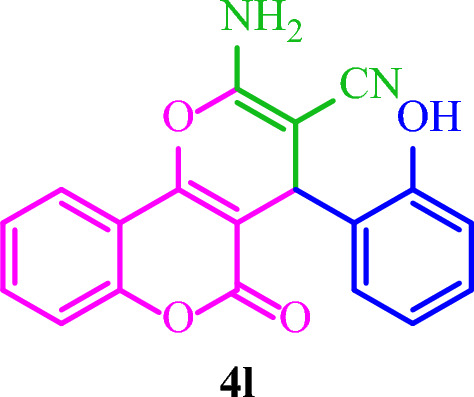


Yellow solid; M.p.: 242–245 °C (reported M.p.: 271–273 °C^39^); IR (KBr): $\upsilon$ (cm^−1^) = 3370, 3195, 3077, 2209, 1701, 1611, 1391, 1043, 757.

**2-Amino-4-(3-ethoxy-4-hydroxyphenyl)-5-oxo-4** ***H*** **,5** ***H*** **-pyrano[3,2-** ***c*** **]chromene-3-carbo- nitrile (4m)** 
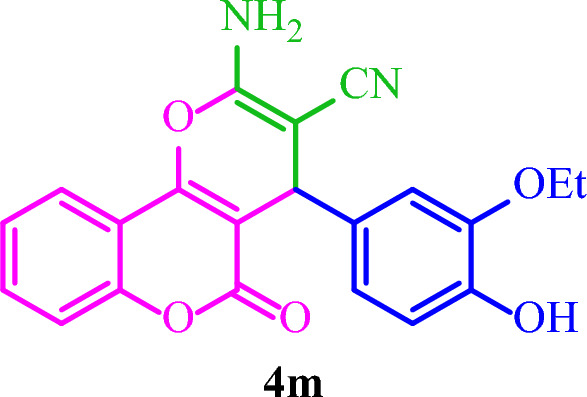


Yellow solid; M.p.: 239–242 °C (reported M.p.: 245–247 °C^36^); IR (KBr): $\upsilon$ (cm^−1^) = 3422, 3320, 3218, 2192, 1685, 1660, 1376, 1049, 753.

## Results and discussion

### Characterization of Basu-proline

The Basu-proline framework was characterized by different following techniques.

#### Characterization by FT‐IR

**Figure 1:**
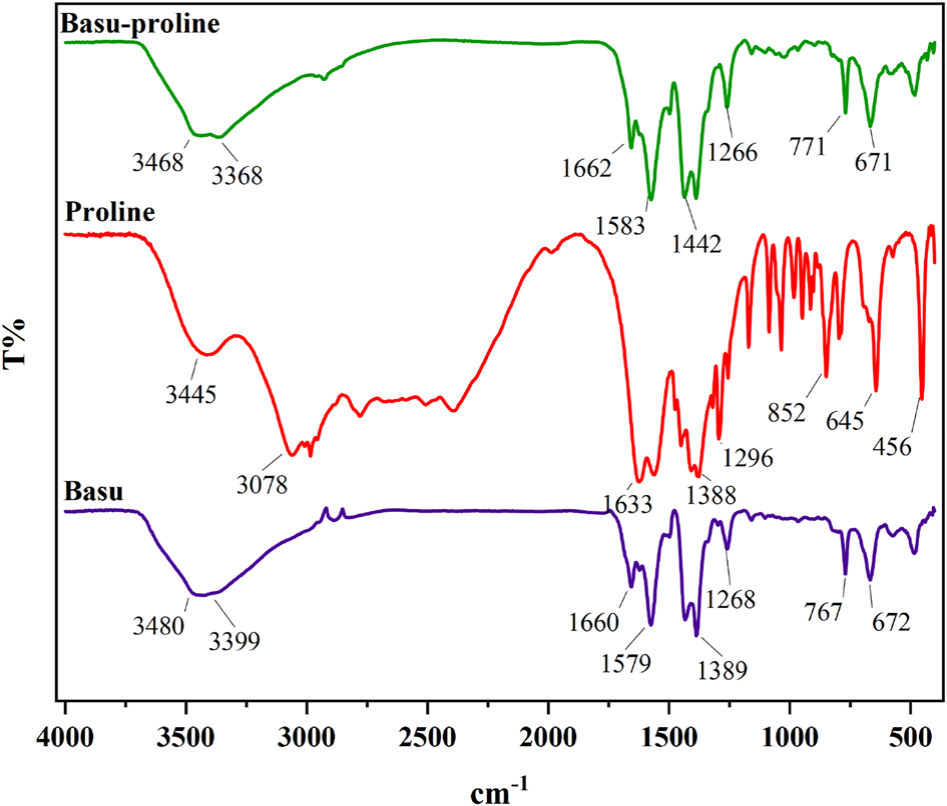
FT-IR spectra of Basu-proline.

The FT-IR spectra of Basu, L-proline, and Basu-proline are exhibited in Fig. 1.

In Basu, the two peaks in 3480 and 3399 cm^−1^ are attributed to the NH_2_ group. The band at 1660 cm^−1^ is probably related to the stretching vibration of the carboxylate group (BDC-NH_2_ ligand). Moreover, the peaks at 1579, 1389, 1268, and 767 cm^−1^ correspond to the stretching vibrations of aromatic C–C, C–O, C_aromatic_–N, and Zr–O bonds, respectively.

In L-proline, the band at 3445 cm^−1^ is attributed to the O–H stretching vibrations of the –COOH group.

In Basu-proline, the two peaks at 3468 and 3368 cm^−1^ are attributed to the NH_2_ group, which is covered by a widening O–H peak due to additional hydrogen bonds. The peak observed at 2802 cm^−1^ belongs to the C–H (sp^3^) bond. Other peaks observed in Basu and L-proline also can be seen in Basu-proline with a slight shift.

#### Characterization by EDX and elemental mapping analysis

**Figure 2:**
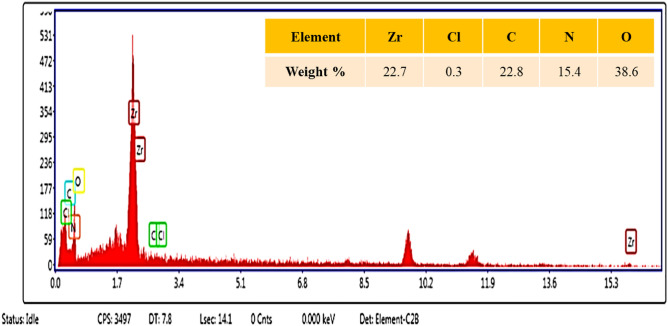
EDX analysis of Basu-proline.

The EDX analysis was performed for the chemical composition characterization of the Basu-proline. As indicated in Fig. 2, the results confirm the presence of Zr, Cl, C, N, and O elements in the porous Basu-proline catalyst.

**Figure 3:**
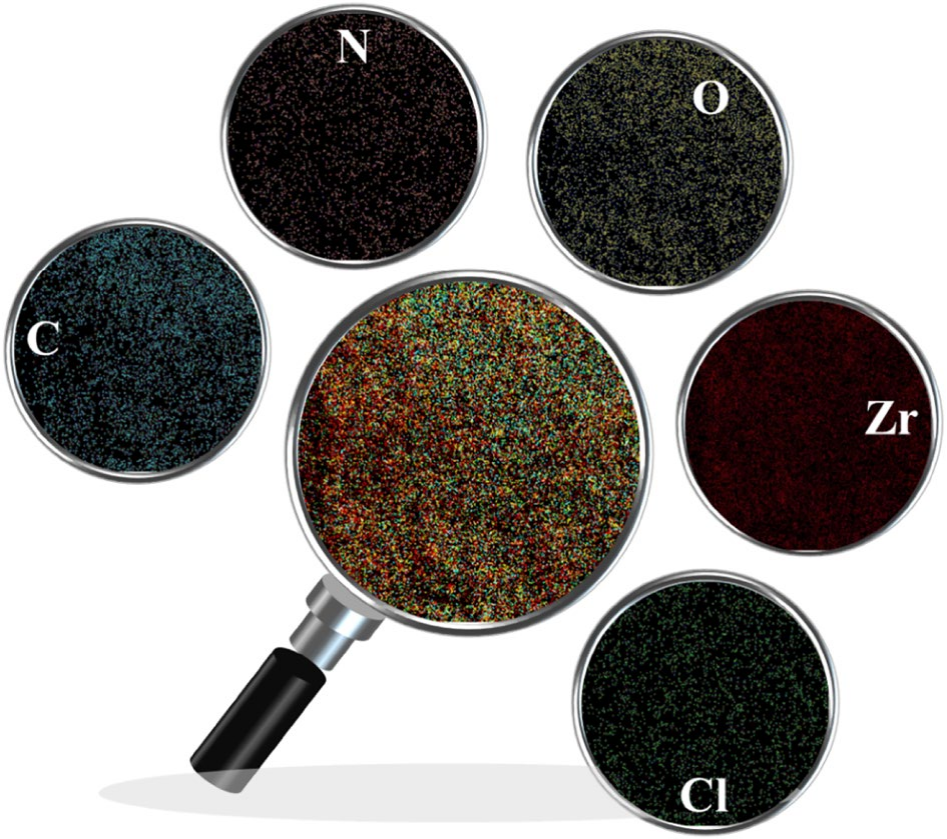
The elemental mapping images of Basu-proline.

The obtained images from the elemental mapping analysis (Fig. 3) confirm the EDX patterns.

#### Characterization by the FE-SEM images

**Figure 4:**
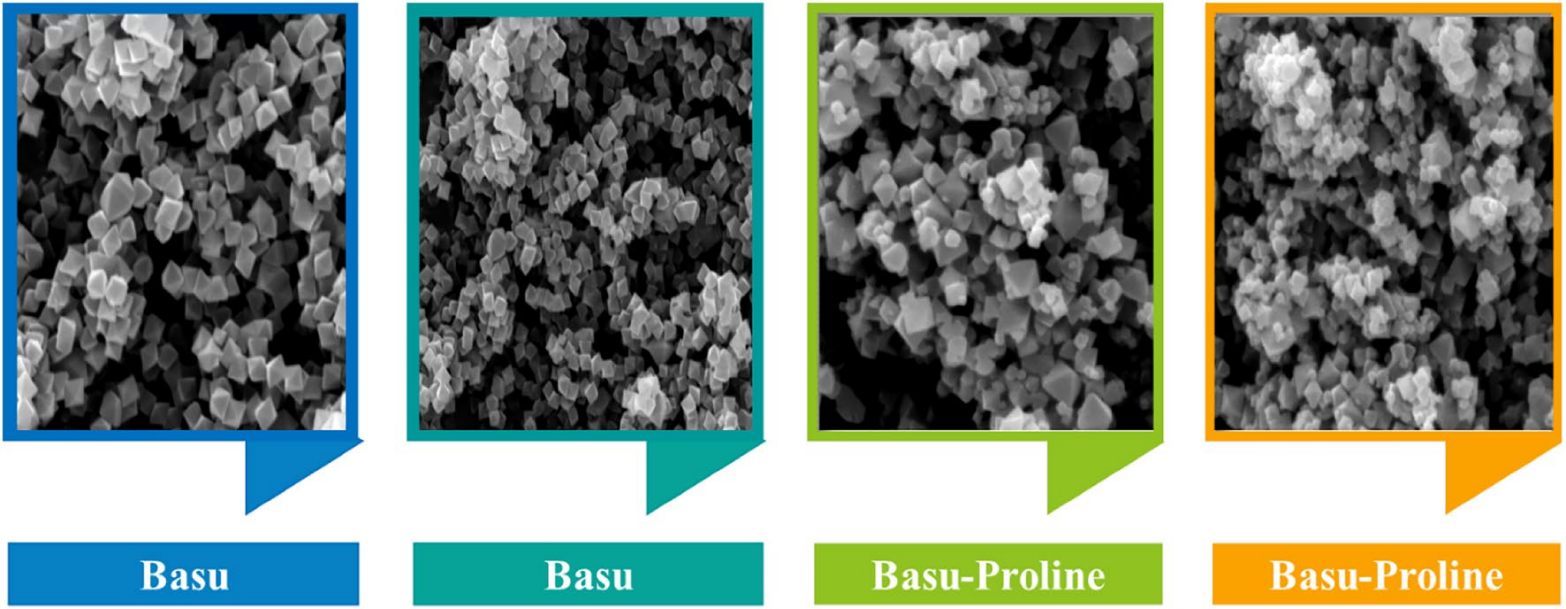
The FE-SEM images of the Basu and Basu-proline.

The structural morphology of the Basu and the Basu-proline were studied by the FE-SEM images (Fig. 4). They show that the Basu framework has an octahedral structure and the particle sizes are uniform and well-distributed homogeneously. Also, the FE-SEM images of Basu-proline show that the octahedral crystalline structure of the Basu was preserved after its post-modification with L-proline.

#### Characterization by XRD

**Figure 5:**
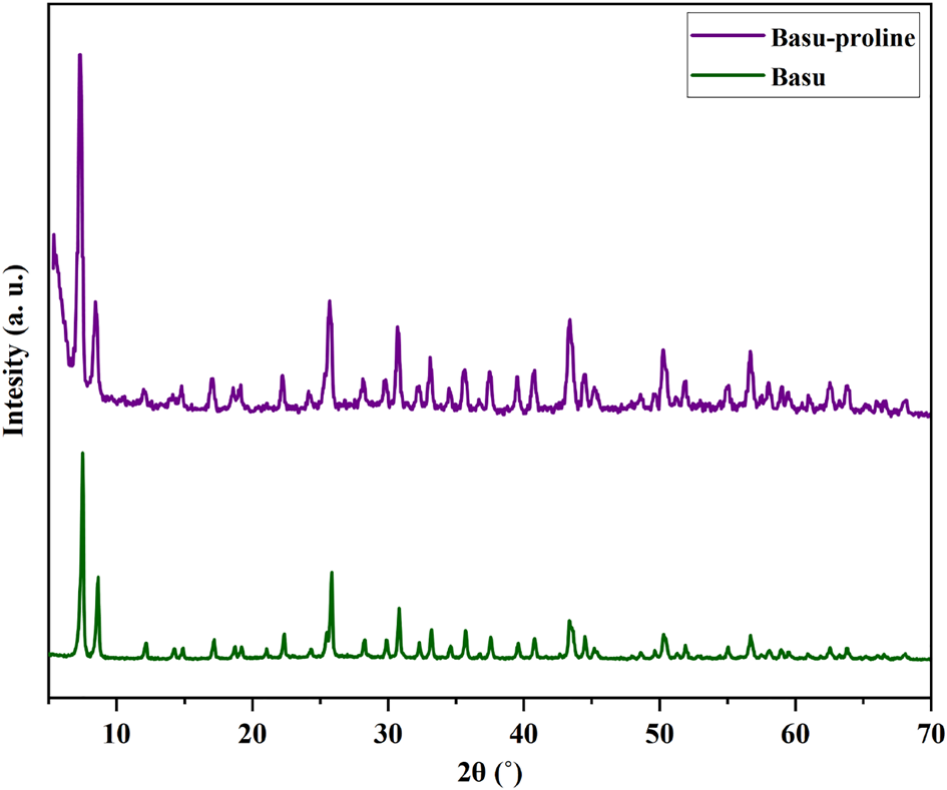
The XRD patterns of Basu and Basu-proline.

In another study, the crystalline phase of the Basu and Basu-proline were determined using XRD analyses (Fig. 5). The typical diffraction peaks of Basu-proline were observed at 2θ = 7.2, 8.4, 17, 22, 25.6, 30.6, 43.3, 50.2, and 56.6, respectively. The achieved pattern is in good agreement with the characteristic peaks of Basu that show the preservation of the crystalline structure during the functionalization and the successful synthesis of the Basu-proline framework.

#### Characterization by N_2_ adsorption–desorption isotherms

**Figure 6:**
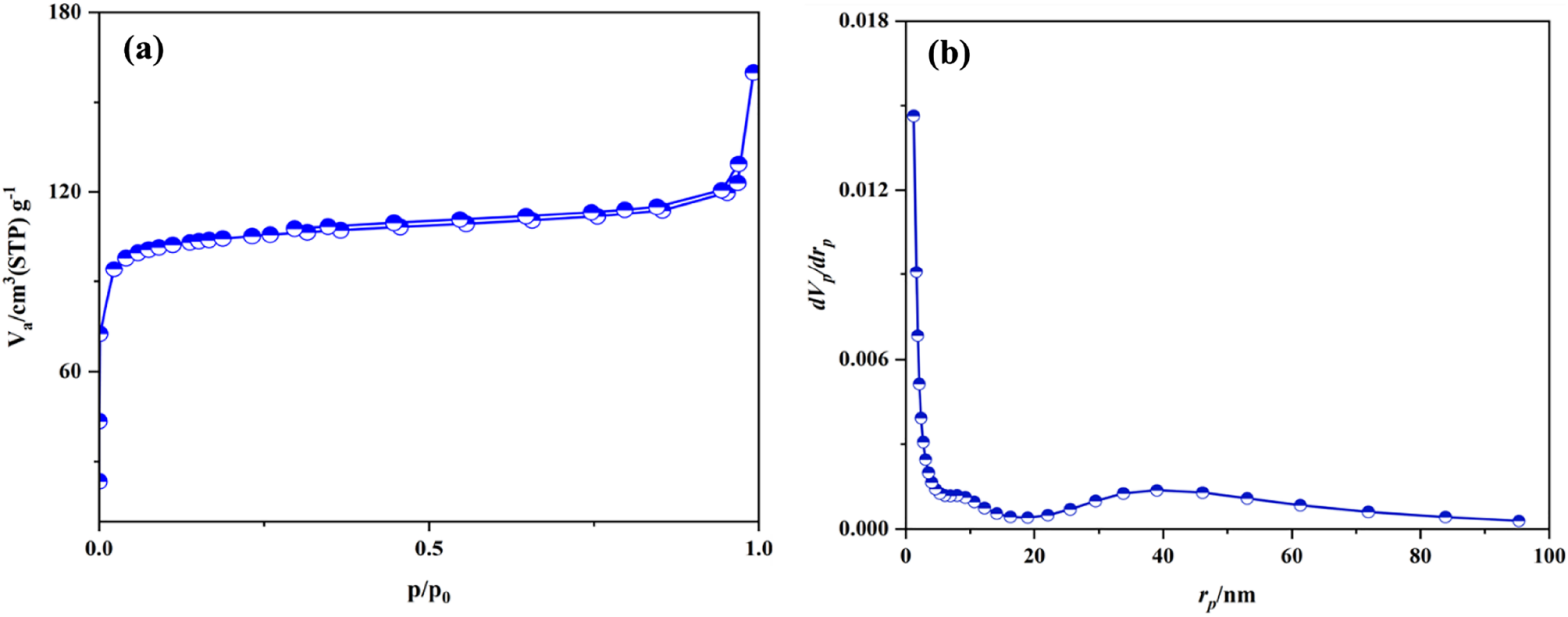
(**a**) The N_2_ adsorption–desorption curve and (**b**) the BJH pore size distribution of Basu-proline.

The N_2_ adsorption–desorption analysis of Basu-proline was performed to identify the surface area, pore volume, and total pore volume. As shown in Fig. 6a, it is like the type II adsorption isotherms based on the IUPAC classification^40^, and the achieved data is depicted in Table 1. Following the post-modification, the surface area and pore volume of Basu-proline compared to the Basu was reduced remarkably to 410.4 m^2^/g and 94.3 cm^3^/g, respectively, which is related to the formation of the larger amide tags than the amine groups.

**Table 1:** The results of N_2_ adsorption–desorption isotherms of Basu and Basu-proline frameworks.

Basu-proline	Basu	Parameter	No
410.4	1107.1	a_s_* (m^2^/g)	1
94.3	254.3	V_m_* (cm^3^/g)	2
0.2	0.8	Total pore volume (cm^3^/g)	3
2.3	2.9	Mean pore diameter (nm)	4

Conditions: N_2_ gas, 77.0 [K], 2 h.

*as: Surface area, by BET method; Vm (pore volume) by BJH method.

Figure 6b demonstrates the BJH adsorption curve of the Basu-proline, which shows that the pore size is about 2.3 nm.

#### Characterization by TGA-DTA

**Figure 7:**
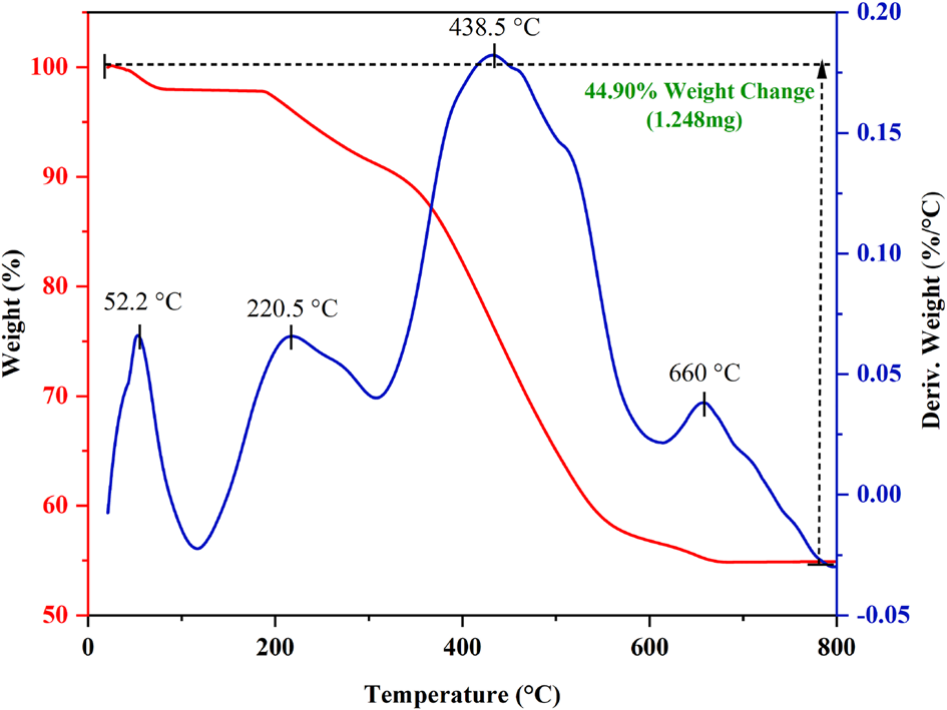
The TGA-DTA curve of the Basu-proline framework.

In another examination, the thermal decomposition behavior of the Basu-proline framework was evaluated using TGA-DTA under airflow (Fig. 7). The weight loss (⁓ 2–7%) below 300 °C is probably related to the evaporation of physically adsorbed moisture/entrapped solvent and the dehydroxylation of the zirconium clusters, respectively. The weight loss (⁓ 45.3–37.5%) between 310 and 800 °C corresponds to organic moieties and framework decomposition. The amount of the remaining sample was calculated to be 55.1% at 800 °C.

### Optimization of the reaction conditions

After preparation and characterization of the Basu-proline framework, its ability as a catalyst was studied in the three-component condensation reaction. For this purpose, the reaction of 4-hydroxycoumarin, 3,4-dimethoxybenzaldehyde**,** and malononitrile was chosen as a model reaction (producing **4a**), and the effect of the mole ratio of starting materials, catalyst loading, solvent, and temperature was studied (Table 2). The best result was found to be a 1:1:1 mol ratio of 4-hydroxycoumarin, 3,4-dimethoxybenzaldehyde**,** and malononitrile with 20 mg of the Basu-proline catalyst in reflux ethanol.

**Table 2:** Optimization of the reaction conditions for the synthesis of 4a.


Entry	Condition	Catalyst amount (mg)	Time (min)	Yield (%)
1	EtOH, reflux	–	35	29
**2**	**EtOH, reflux**	**20**	**35**	**94**
3	EtOH, reflux	40	35	88
4	EtOH, reflux	60	35	68
5	EtOH:H_2_O, 80 °C	20	35	40
6	CH_3_CN, reflux	20	35	51
**7**	CHCl_3_, reflux	20	35	40
8	CH_2_Cl_2_, reflux	20	35	30
9	Solvent-free, 110 °C	20	35	55

Significant values are in bold.

Increasing the temperature and amount of the catalyst did not affect the reaction efficiency.

In another study, the performance of the Basu-proline catalyst was compared with several known catalysts (Table 3), indicating that those reported catalysts have weaker performances than Basu-proline.

**Table 3:** Screening the model reaction in the presence of reported known catalysts.

Entry	Catalyst	Catalyst amount (mg)	Time (min)	Yield (%)
1	L-Proline	20	50	37
2	Basu	20	180	67
3	Piperidine	20	120	27
4	Triethylamine	20	160	27
5	*p*-TSA	20	45	53
6	Zr-UiO-66-PDC	20	50	63
7	UiO-66-NH_2_	20	35	42

### Synthesis of diverse dihydro-pyrano[3,2-*c*]chromenes 4(a-m)

Based on the optimal reaction conditions, a wide range of aromatic aldehydes bearing both electron‐donating and electron‐withdrawing groups were reacted with 4-hydroxycoumarin and malononitrile by Basu-proline in reflux conditions to yield dihydro-pyrano[3,2-*c*]chromenes **4(a-m)** in short reaction times and good yields (Table 4). The desired products were then purified by washing with ethanol.

**Table 4:**
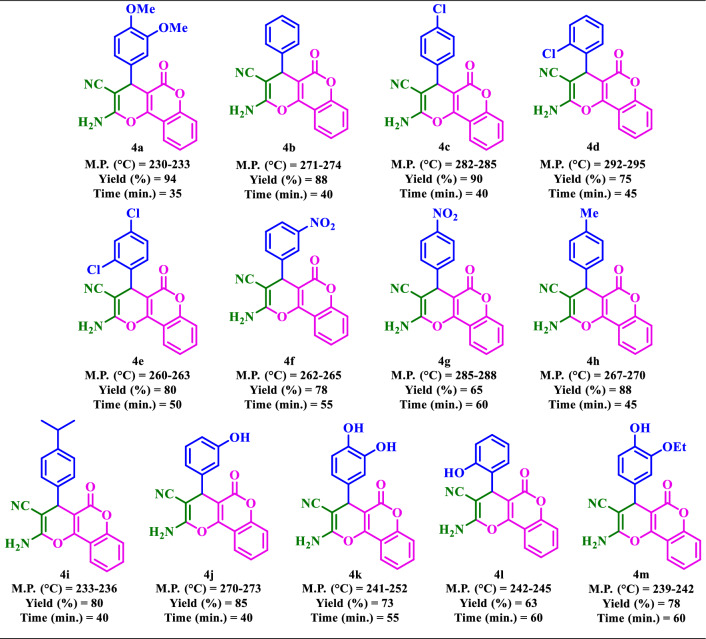
Synthesis of diverse dihydro-pyrano[3,2-*c*]chromenes **4(a-m)** by the Basu-proline catalyst^a^.

^**a**^Reaction conditions: 4-hydroxycoumarin (1.0 mmol), malononitrile (1.0 mmol), aldehyde (1.0 mmol), and the Basu-proline catalyst (20 mg) in EtOH (3.0 mL) under reflux conditions.

### A proposed mechanism for the synthesis of dihydro-pyrano[3,2-*c*]chromenes 4(a-m)

**Scheme 3:**
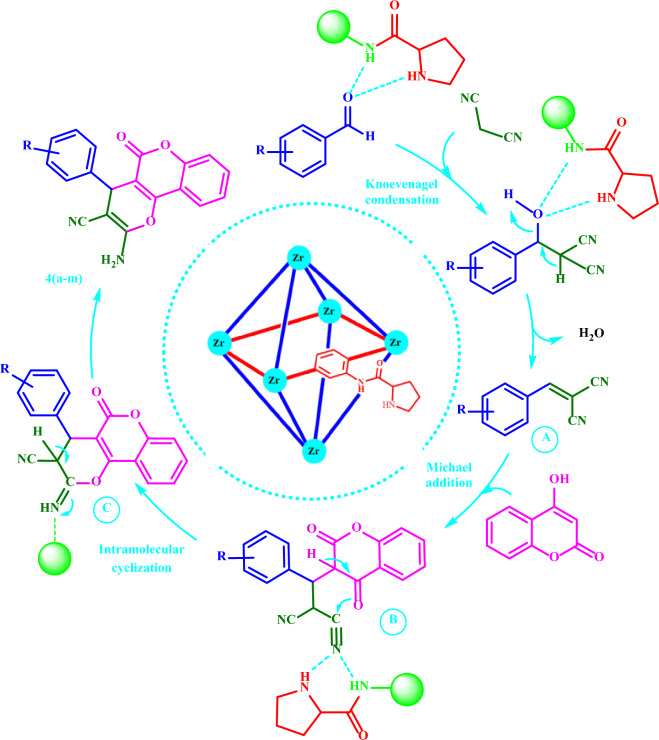
A proposed mechanism for the synthesis of **4(a-m).**

The proposed mechanism for the reaction is presented in Scheme 3. Condensations of the activated aldehyde with malononitrile give the corresponding adducts **A** with the subsequent nucleophilic additions of 4-hydroxycoumarin. Then, the intramolecular cyclization of the resulting adducts **B** affords the related **4(a-m)** in good yields (63–94%).

### Reusability of the Basu-proline Framework

**Figure 8:**
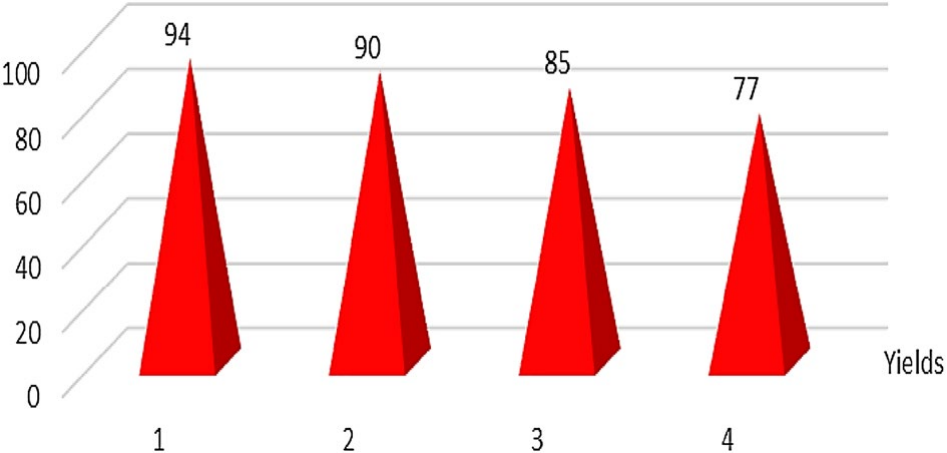
Recycling study of Basu-proline.

Since one of the essential advantages of heterogeneous catalysts is their recovery capability, the recovery and reusability of Basu-proline were also checked in the model reaction. As can be seen in Fig. 8.

The Basu-proline relatively maintains its activity even after four successive runs with a low decrease in its activity.

**Figure 9:**
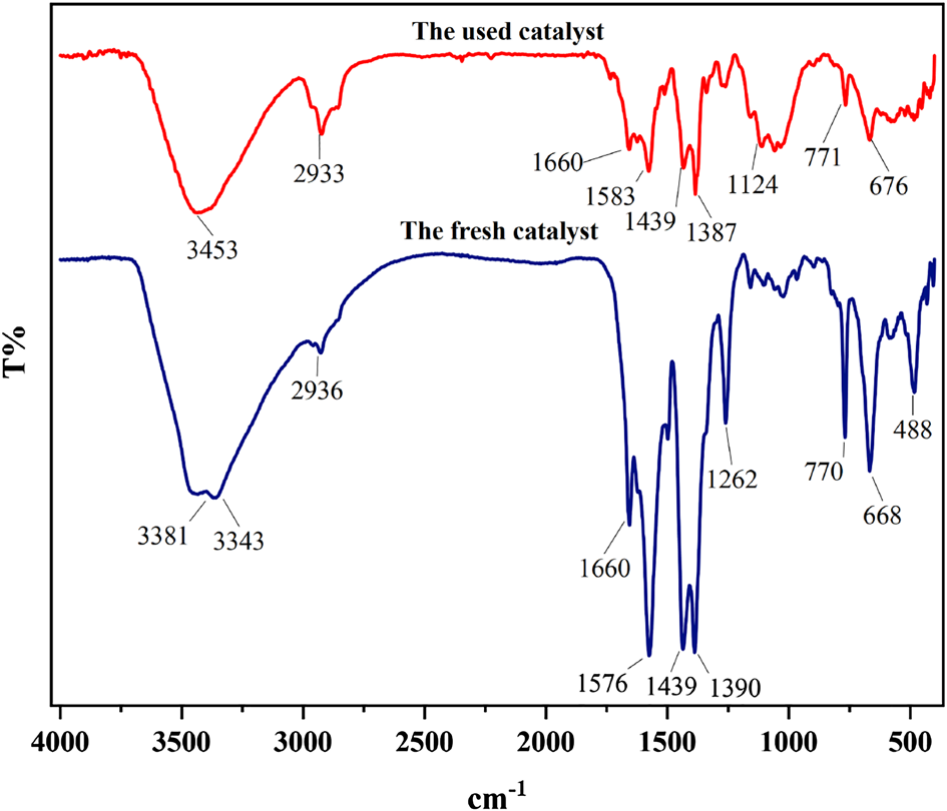
The fresh and used Basu-proline FT-IR spectra.

In addition, the used catalyst was evaluated after the four catalytic cycles by the FT-IR, FE-SEM, and XRD techniques. As demonstrated in Fig. 9, the characteristic peaks of the fresh catalyst were preserved in the used catalyst, which shows the stability of the recycled catalyst.

**Figure 10:**
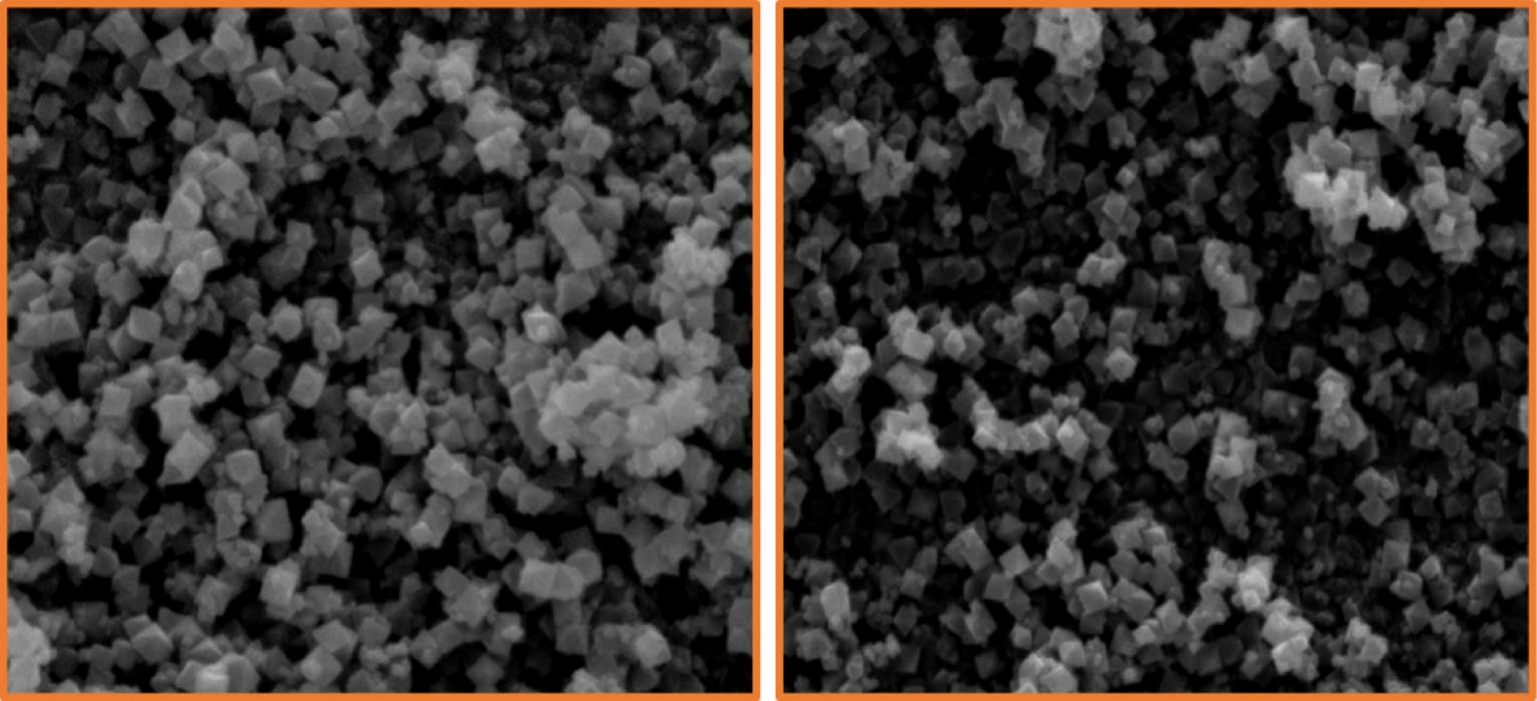
The FE-SEM images of the used Basu-proline catalyst.

**Figure 11:**
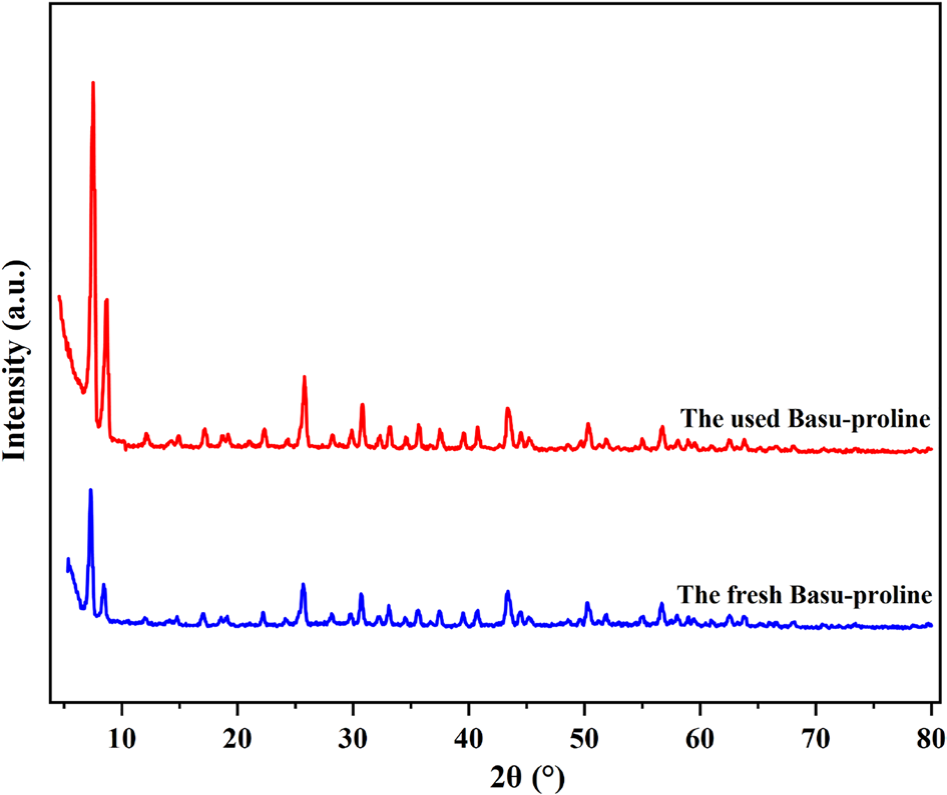
The fresh and used Basu-proline catalyst XRD patterns.

The FE-SEM images also showed (Fig. 10) that the catalyst’s structure remained intact. Moreover, the crystalline phase of the used Basu-proline catalyst has been preserved based on the XRD curve (Fig. 11).

Leaching of the metal catalyst is one of the important challenges for synthetic chemists^41,42^. Hence, a hot filtration test was performed to evaluate the heterogeneous nature of the prepared catalyst. The result obtained from the ICP/MS analysis shows that the Zr leaching (Zr: 15 × 10^–6^ mol/g) upon completion of the reaction is neglectable, indicating the high stability of the catalyst.

### Comparison of the catalyst activities

For further study of the catalytic performance of Basu-proline, the efficiency of the proposed protocol with some previously reported methods was compared. As can be observed in Table 5, the Basu-proline catalyst allows the formation of desired products in less reaction time with high efficiency compared to some previously reported protocols.

**Table 5:** Comparison of the catalyst activities.

	(1) Z-HY@SiO_2_-Pr-Py, solvent-free, 100 °C, 80 min, 92%^22^ (2) 2-Hydroxyethylammonium formate, solvent-free, r.t., 6 min, 68%^27^ (3) Immobilized laccase, TEMPO, aqueous medium, 40 °C, 17 h, 75%^14^ (4) The Basu-proline catalyst, EtOH, reflux, 35 min, 94% [Current work]
	(1) Z-HY@SiO_2_-Pr-Py, solvent-free, 100 °C, 90 min, 95%^22^ (2) H_5_BW_12_O_40_, EtOH/H_2_O, reflux, 270 min, 98%^24^ (3) Urea, EtOH/H_2_O, r.t., 7 h, 93%^18^ (4) Dehydroabietylamine/cinchonine/squaramide, r.t., 24 h, 84%^17^ (5) Tertiary amine-thiourea, Et_2_O, r.t., 12 h, 85%^28^ (6) The Basu-proline catalyst, EtOH, reflux, 40 min, 90% [Current work]
	(1) Z-HY@SiO_2_-Pr-Py, solvent-free, 100 °C, 90 min, 92%^22^ (2) H_5_BW_12_O_40_, EtOH/H_2_O, reflux, 90 min, 88%^24^ (3) Urea, EtOH/H_2_O, r.t., 10 h, 91%^18^ (4) The Basu-proline catalyst, EtOH, reflux, 45 min, 88% [Current work]
	(1) Urea, EtOH/H_2_O, r.t., 3 h, 97%^18^ (2) Z-HY@SiO_2_-Pr-Py, solvent-free, 100 °C , 80 min, 93%^22^ (3) H_5_BW_12_O_40_, EtOH/H_2_O, reflux, 300 min, 85%^24^ (4) The Basu-proline catalyst, EtOH, reflux, 55 min, 78% [Current work]

## Conclusion

Briefly, the present study describes the preparation of the L-proline-modified Zr-based metal–organic framework Basu-proline and its application as a heterogeneous catalyst for the synthesis of diverse dihydro-pyrano[3,2-*c*]chromenes **4(a-m)** via Knoevenagel condensation-Michael addition-intramolecular cyclization tandem sequence under mild conditions. According to the FE-SEM images, the Basu MOF has an octahedron structure that has preserved its structure after modification with L-proline. The advantages of this protocol involve easy work-up, short reaction time, high efficiency, low catalyst loading, reusability of catalyst, recyclability, and compatibility with electron‐donating groups and electron‐withdrawing groups. Generally, a green, efficient, and economical procedure was used to accelerate the synthesis of dihydropyrano[3,2-*c*]chromenes by various aromatic aldehydes with various structural differences (Supplementary file). The prepared catalyst displays high recyclability for four cycles without a remarkable loss in its catalytic performance. The efficient and green synthesis of dihydropyrano[3,2-*c*]chromenes with the assistance of a Basu-proline catalyst in EtOH indicates the promising applications of Basu-proline in synthesizing heterocycle compounds.

### Supplementary Information

Supplementary Information.

## Data Availability

All data generated or analyzed during this study are included in this published article and its supplementary information file.
