# Supplementary material for: The L-proline modified Zr-based MOF (Basu-proline) catalyst for the one‐pot synthesis of dihydropyrano[3,2-c]chromenes
Source: Sci Rep. 2023 Oct 17;13:17608. doi: 10.1038/s41598-023-44774-4 (PMC10582120; doi:10.1038/s41598-023-44774-4)
Supplement: Supplementary file 1 — Supplementary Information. [file 41598_2023_44774_MOESM1_ESM.docx]

**Supporting Information**

(Scientific Reports)

A Zr-based MOF (Basu-proline) catalyst for the one‐pot synthesis of dihydropyrano[3,2-*c*]chromenes via a tandem three‐component reaction

**Amin Benrashid, Davood Habibi*, Masoumeh Beiranvand,** [**Maryam Mahmoudiani Gilan**](https://onlinelibrary.wiley.com/authored-by/Mahmoudiani+Gilan/Maryam)

Department of Organic Chemistry, Faculty of Chemistry, Bu-Ali Sina University, Hamedan, Iran

*Corresponding author email: [davood.habibi@gmail.com](mailto:davood.habibi@gmail.com) (& dhabibi@basu.ac.ir), Tel: +98 81 38380922;

Fax: +98 81 38380709

Contents Page

[FT-IR Spectrum of *2-Amino-4-(3,4-dimethoxyphenyl)-5-oxo-4H,5H-pyrano[3,2-c]chromene-3-carbonitrile* (**4a**) 1](#_Toc147673601)

[^1^H NMR Spectrum of *2-Amino-4-(3,4-dimethoxyphenyl)-5-oxo-4H,5H-pyrano[3,2-c]chromene-3-carbonitrile* (**4a**) 1](#_Toc147673602)

[^13^C NMR Spectrum of *2-Amino-4-(3,4-dimethoxyphenyl)-5-oxo-4H,5H-pyrano[3,2-c]chromene-3-carbonitrile* (**4a**) 2](#_Toc147673603)

[FT-IR Spectrum of *2-Amino-5-oxo-4-phenyl-4H,5H-pyrano[3,2-c]chromene-3-carbonitrile* (**4b**) 2](#_Toc147673604)

[^1^H NMR Spectrum of *2-Amino-5-oxo-4-phenyl-4H,5H-pyrano[3,2-c]chromene-3-carbonitrile* (**4b**) 3](#_Toc147673605)

[^13^C NMR Spectrum of *2-Amino-5-oxo-4-phenyl-4H,5H-pyrano[3,2-c]chromene-3-carbonitrile* (**4b**) 4](#_Toc147673606)

[FT-IR Spectrum of *2-Amino-4-(4-chlorophenyl)-5-oxo-4H,5H-pyrano[3,2-c]chromene-3-carbonitrile* (**4c**) 4](#_Toc147673607)

[^1^H NMR Spectrum of *2-Amino-4-(4-chlorophenyl)-5-oxo-4H,5H-pyrano[3,2-c]chromene-3-carbonitrile* (**4c**) 5](#_Toc147673608)

[^13^C NMR Spectrum of *2-Amino-4-(4-chlorophenyl)-5-oxo-4H,5H-pyrano[3,2-c]chromene-3-carbonitrile* (**4c**) 6](#_Toc147673609)

[FT-IR Spectrum of *2-Amino-4-(2-chlorophenyl)-5-oxo-4H,5H-pyrano[3,2-c]chromene-3-carbonitrile* (**4d**) 6](#_Toc147673610)

[^1^H NMR Spectrum of *2-Amino-4-(2-chlorophenyl)-5-oxo-4H,5H-pyrano[3,2-c]chromene-3-carbonitrile* (**4d**) 7](#_Toc147673611)

[^13^C NMR Spectrum of *2-Amino-4-(2-chlorophenyl)-5-oxo-4H,5H-pyrano[3,2-c]chromene-3-carbonitrile* (**4d**) 8](#_Toc147673612)

[FT-IR Spectrum of *2-Amino-4-(2,4-dichlorophenyl)-5-oxo-4H,5H-pyrano[3,2-c]chromene-3-carbonitrile* (**4e**) 8](#_Toc147673613)

[^1^H NMR Spectrum of *2-Amino-4-(2,4-dichlorophenyl)-5-oxo-4H,5H-pyrano[3,2-c]chromene-3-carbonitrile* (**4e**) 9](#_Toc147673614)

[^13^C NMR Spectrum of *2-Amino-4-(2,4-dichlorophenyl)-5-oxo-4H,5H-pyrano[3,2-c]chromene-3-carbonitrile* (**4e**) 10](#_Toc147673615)

[FT-IR Spectrum of *2-Amino-4-(3-nitrophenyl)-5-oxo-4H,5H-pyrano[3,2-c]chromene-3-carbonitrile* (**4f**) 10](#_Toc147673616)

[^1^H NMR Spectrum of *2-Amino-4-(3-nitrophenyl)-5-oxo-4H,5H-pyrano[3,2-c]chromene-3-carbonitrile* (**4f**) 11](#_Toc147673617)

[^13^C NMR Spectrum of *2-Amino-4-(3-nitrophenyl)-5-oxo-4H,5H-pyrano[3,2-c]chromene-3-carbonitrile* (**4f**) 12](#_Toc147673618)

[FT-IR Spectrum of *2-Amino-4-(4-nitrophenyl)-5-oxo-4H,5H-pyrano[3,2-c]chromene-3-carbonitrile* (**4g**) 12](#_Toc147673619)

[FT-IR Spectrum of *2-Amino-5-oxo-4-(p-tolyl)-4H,5H-pyrano[3,2-c]chromene-3-carbonitrile* (**4h**) 13](#_Toc147673620)

[FT-IR Spectrum of *2-Amino-4-(4-isopropylphenyl)-5-oxo-4H,5H-pyrano[3,2-c]chromene-3-carbonitrile* (**4i**) 13](#_Toc147673621)

[FT-IR Spectrum *2-Amino-4-(3-hydroxyphenyl)-5-oxo-4H,5H-pyrano[3,2-c]chromene-3-carbonitrile* (**4j**) 14](#_Toc147673622)

[FT-IR Spectrum of *2-Amino-4-(2,3-dihydroxyphenyl)-5-oxo-4H,5H-pyrano[3,2-c]chromene-3-carbonitrile* (**4k**) 14](#_Toc147673623)

[FT-IR Spectrum of *2-Amino-4-(2-hydroxyphenyl)-5-oxo-4H,5H-pyrano[3,2-c]chromene-3-carbonitrile* (**4l**) 15](#_Toc147673624)

[FT-IR Spectrum of *2-Amino-4-(3-ethoxy-4-hydroxyphenyl)-5-oxo-4H,5H-pyrano[3,2-c] chromene-3-carbo nitrile* (**4m**) 15](#_Toc147673625)


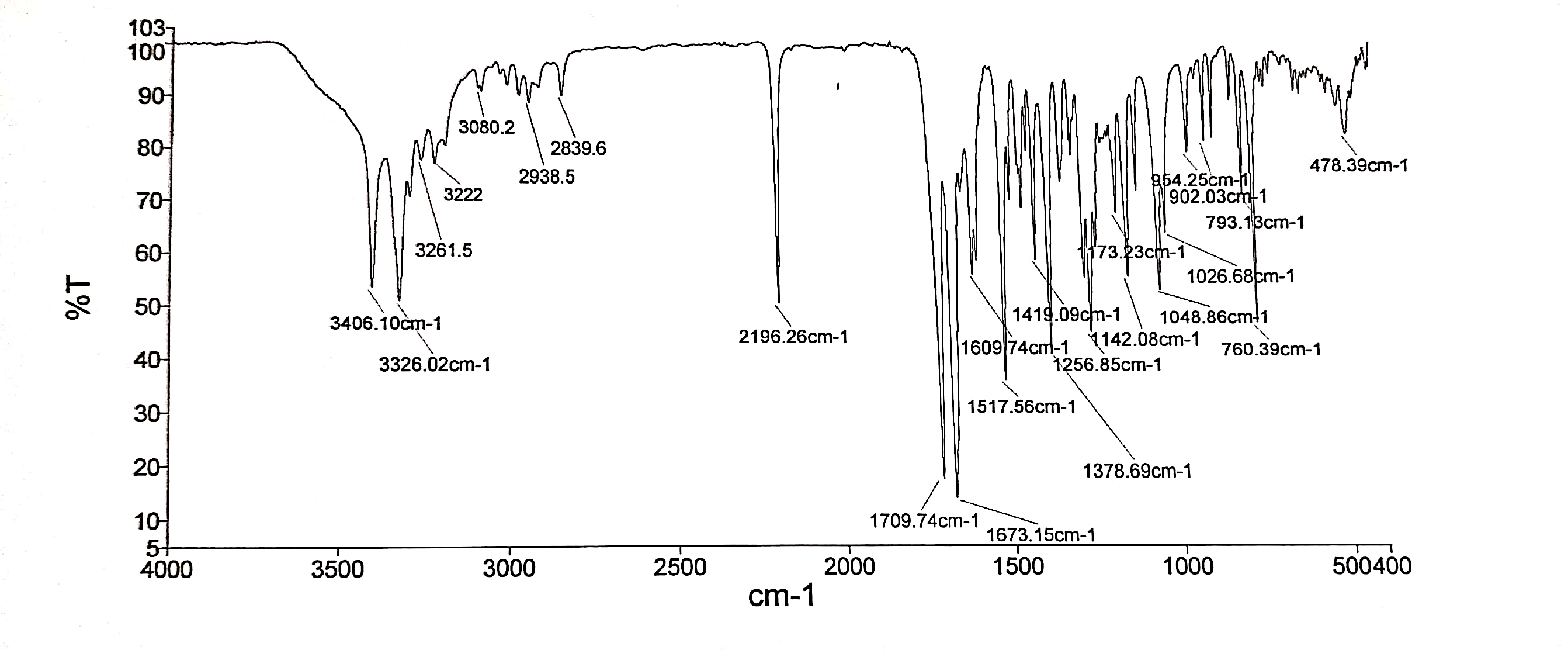


FT-IR Spectrum of *2-Amino-4-(3,4-dimethoxyphenyl)-5-oxo-4H,5H-pyrano[3,2-c]chromene-3-carbonitrile* (**4a**)


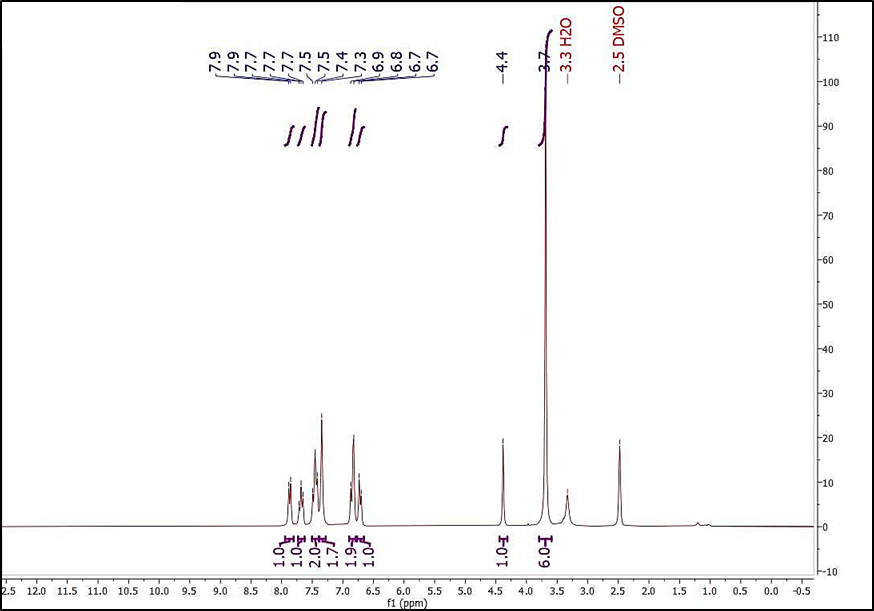


^1^H NMR Spectrum of *2-Amino-4-(3,4-dimethoxyphenyl)-5-oxo-4H,5H-pyrano[3,2-c]chromene-3-carbonitrile* (**4a**)


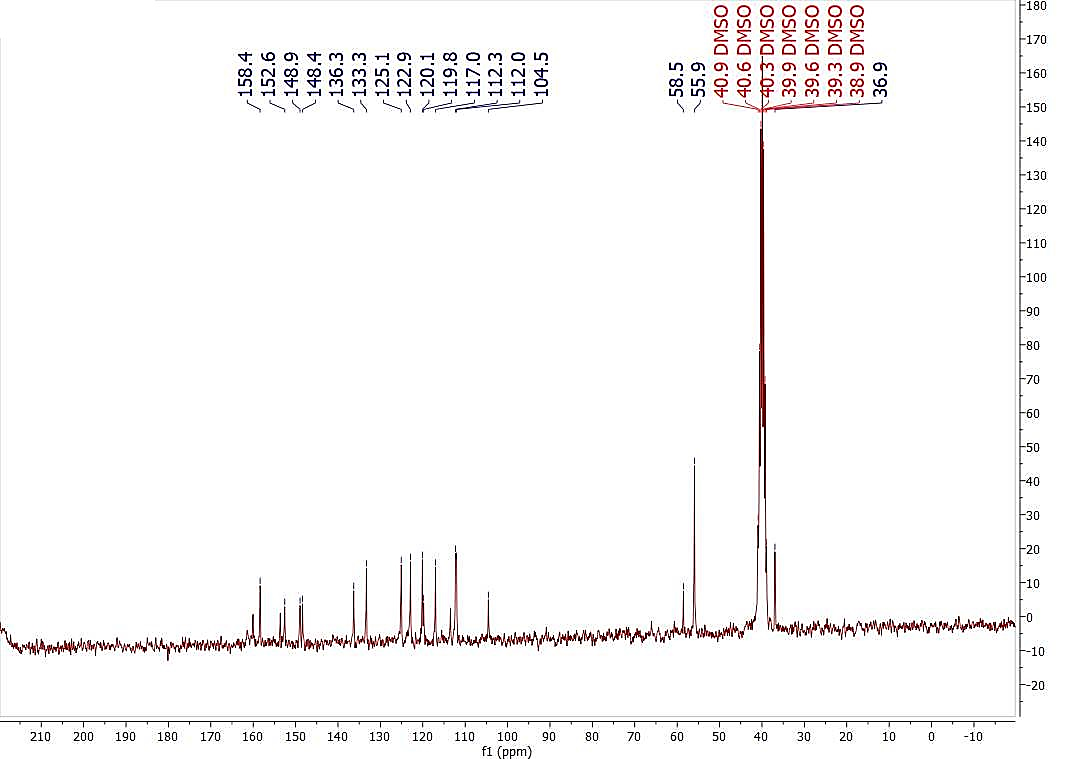


^13^C NMR Spectrum of *2-Amino-4-(3,4-dimethoxyphenyl)-5-oxo-4H,5H-pyrano[3,2-c]chromene-3-carbonitrile* (**4a**)


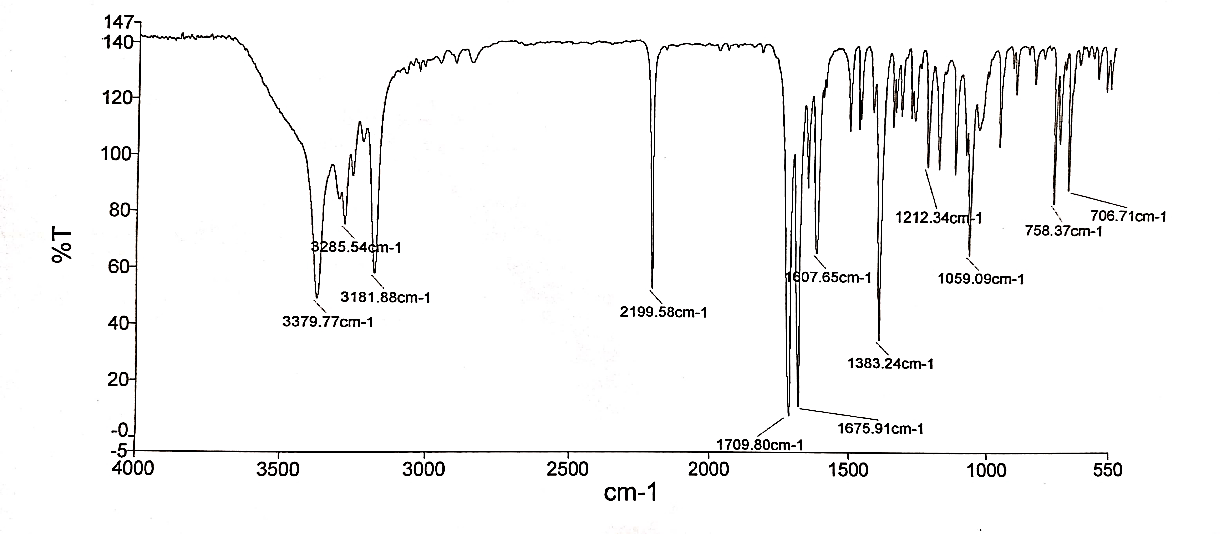


FT-IR Spectrum of *2-Amino-5-oxo-4-phenyl-4H,5H-pyrano[3,2-c]chromene-3-carbonitrile* (**4b**)


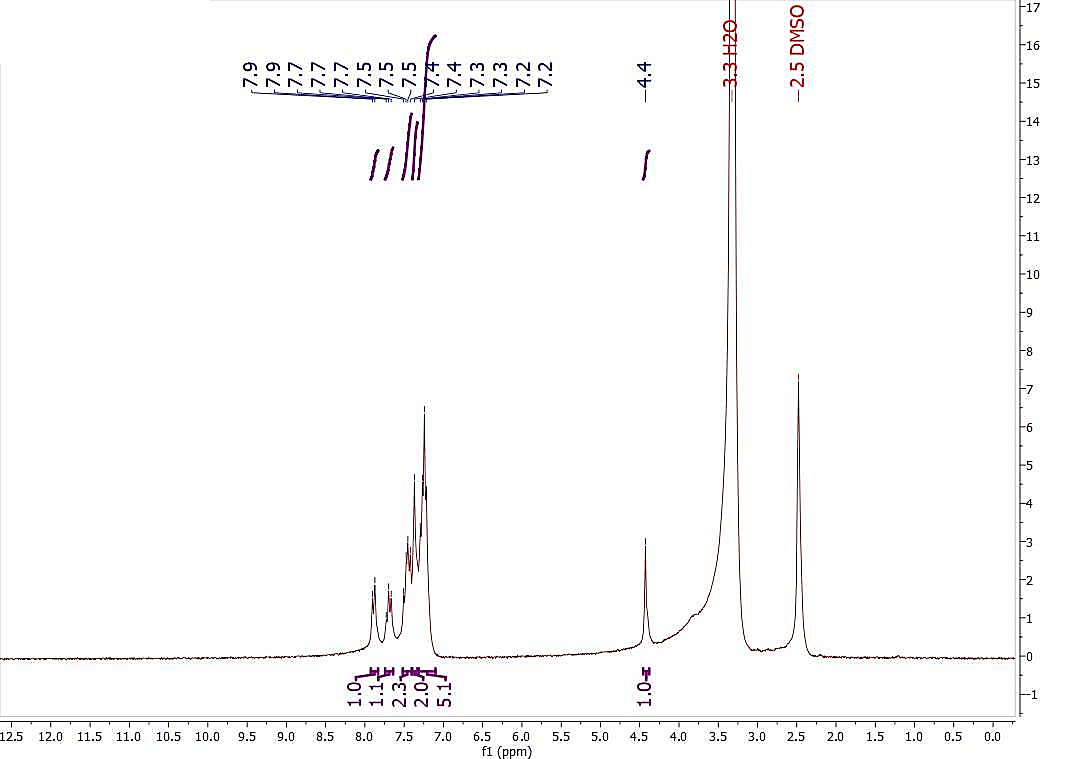


^1^H NMR Spectrum of *2-Amino-5-oxo-4-phenyl-4H,5H-pyrano[3,2-c]chromene-3-carbonitrile* (**4b**)


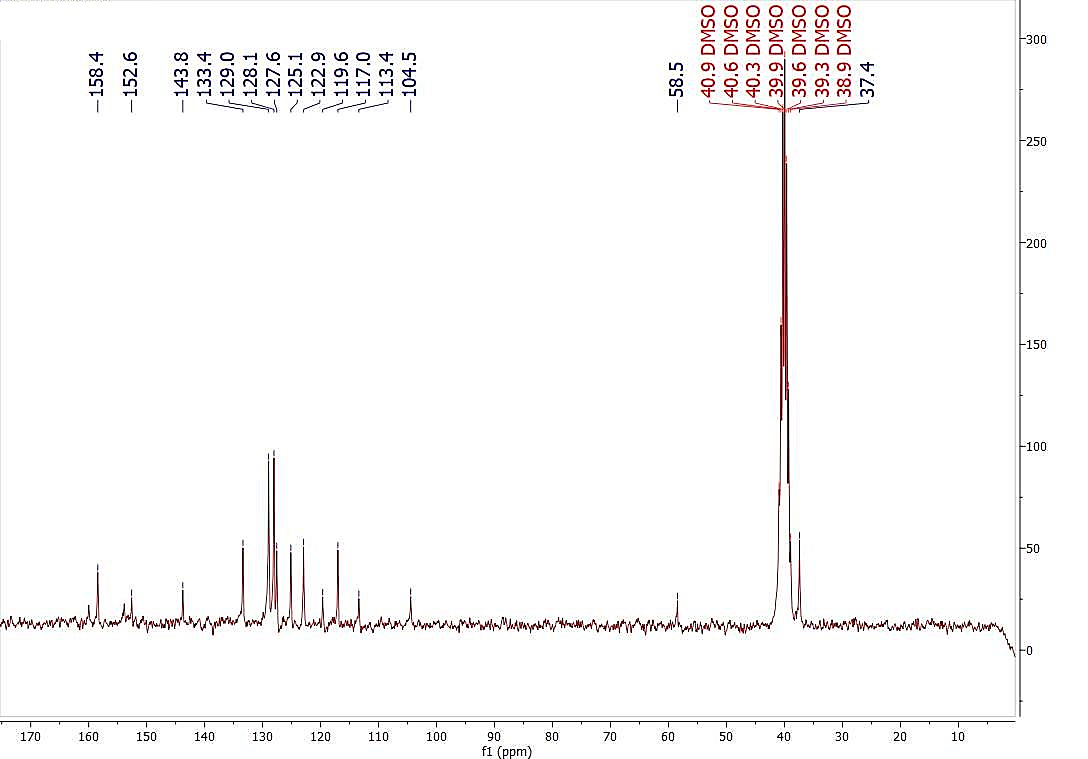


^13^C NMR Spectrum of *2-Amino-5-oxo-4-phenyl-4H,5H-pyrano[3,2-c]chromene-3-carbonitrile* (**4b**)


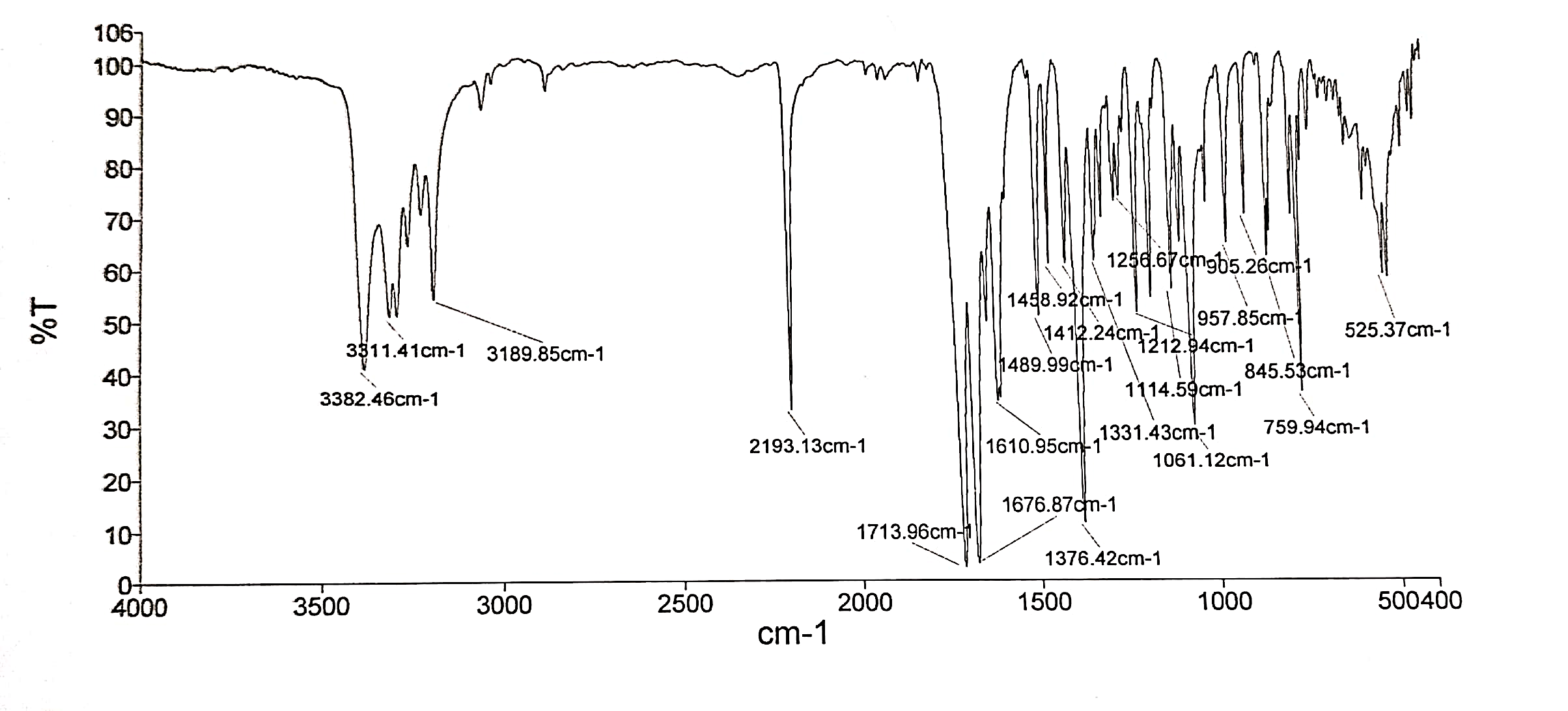


FT-IR Spectrum of *2-Amino-4-(4-chlorophenyl)-5-oxo-4H,5H-pyrano[3,2-c]chromene-3-carbonitrile* (**4c**)


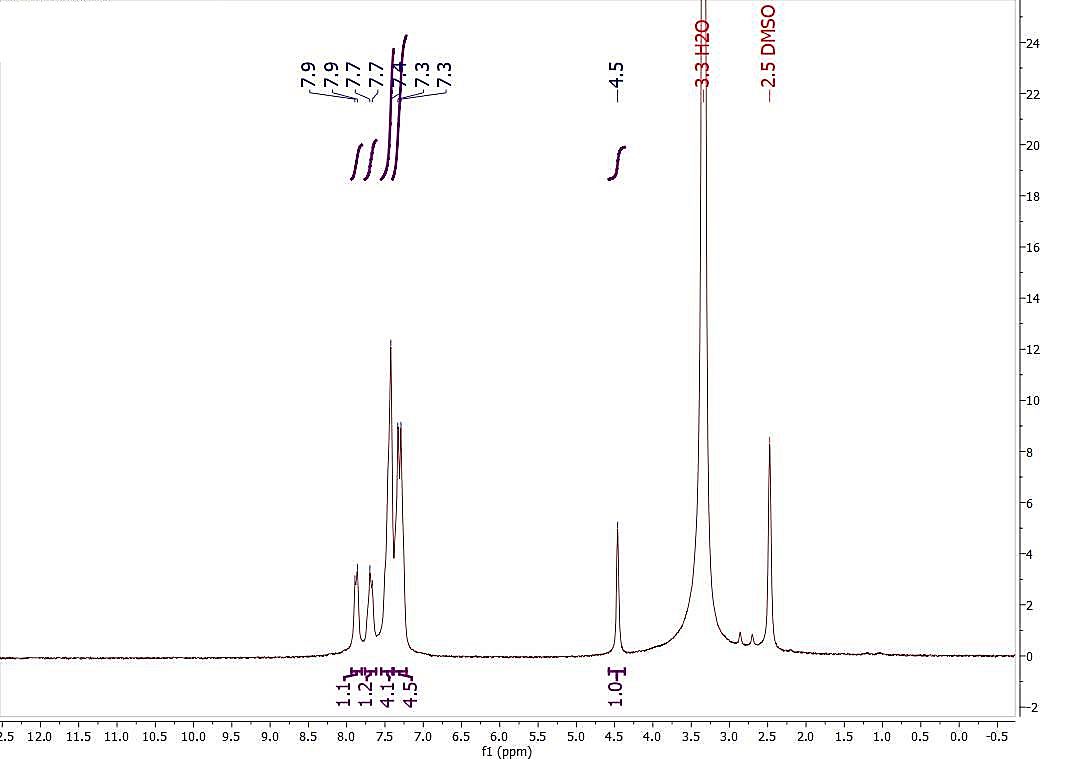


^1^H NMR Spectrum of *2-Amino-4-(4-chlorophenyl)-5-oxo-4H,5H-pyrano[3,2-c]chromene-3-carbonitrile* (**4c**)


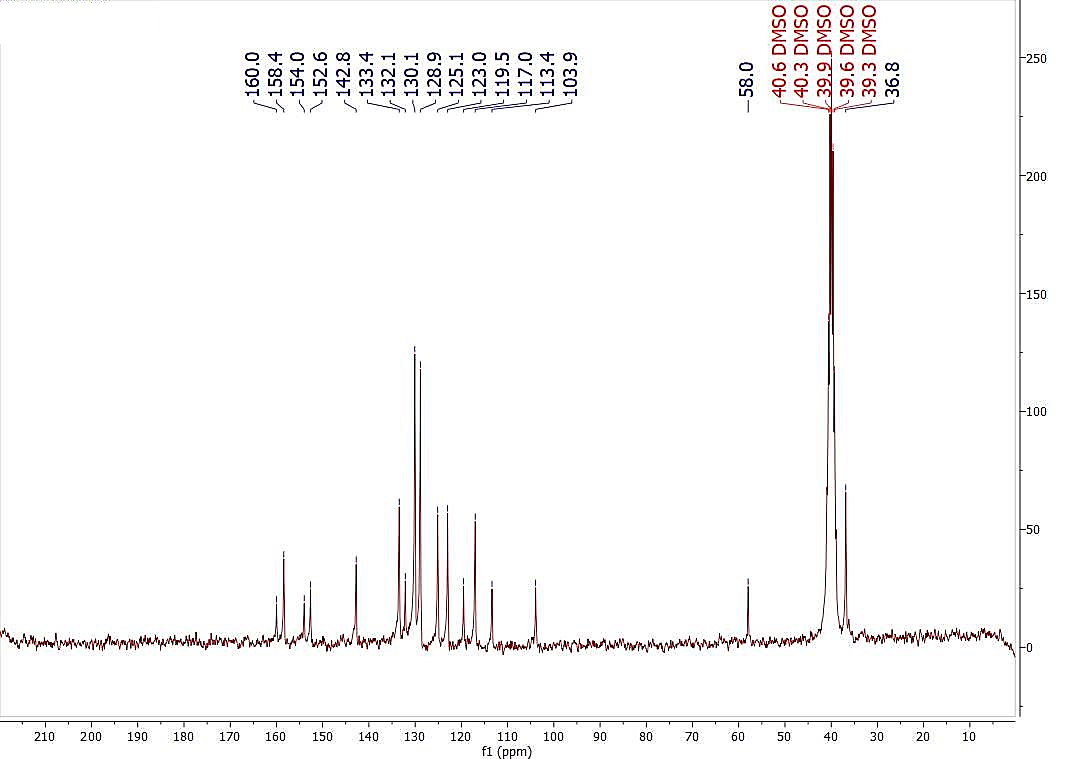


^13^C NMR Spectrum of *2-Amino-4-(4-chlorophenyl)-5-oxo-4H,5H-pyrano[3,2-c]chromene-3-carbonitrile* (**4c**)


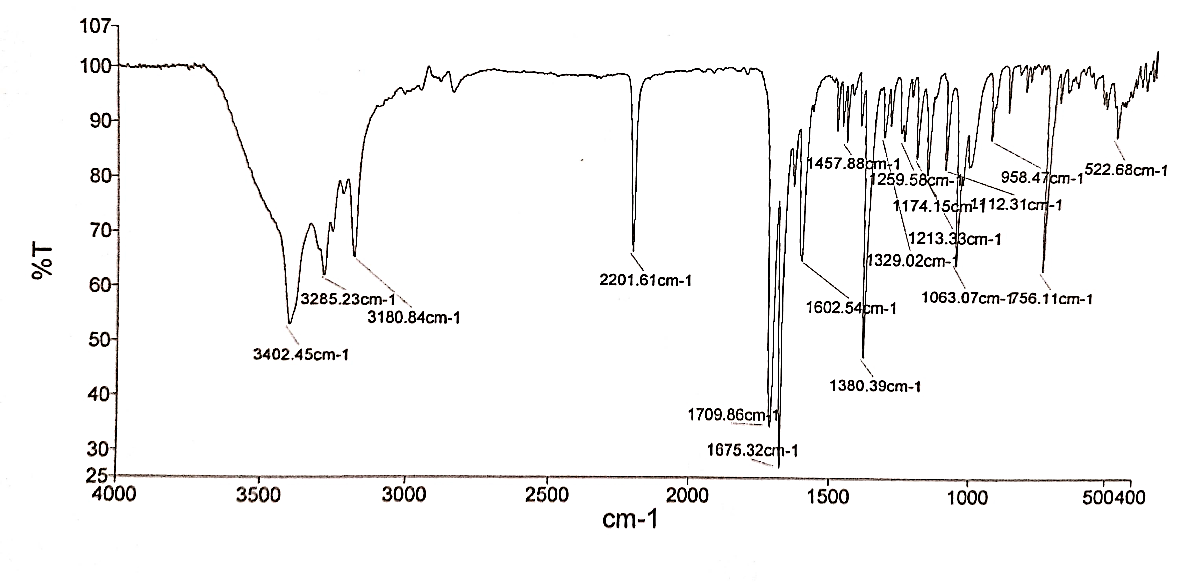


FT-IR Spectrum of *2-Amino-4-(2-chlorophenyl)-5-oxo-4H,5H-pyrano[3,2-c]chromene-3-carbonitrile* (**4d**)


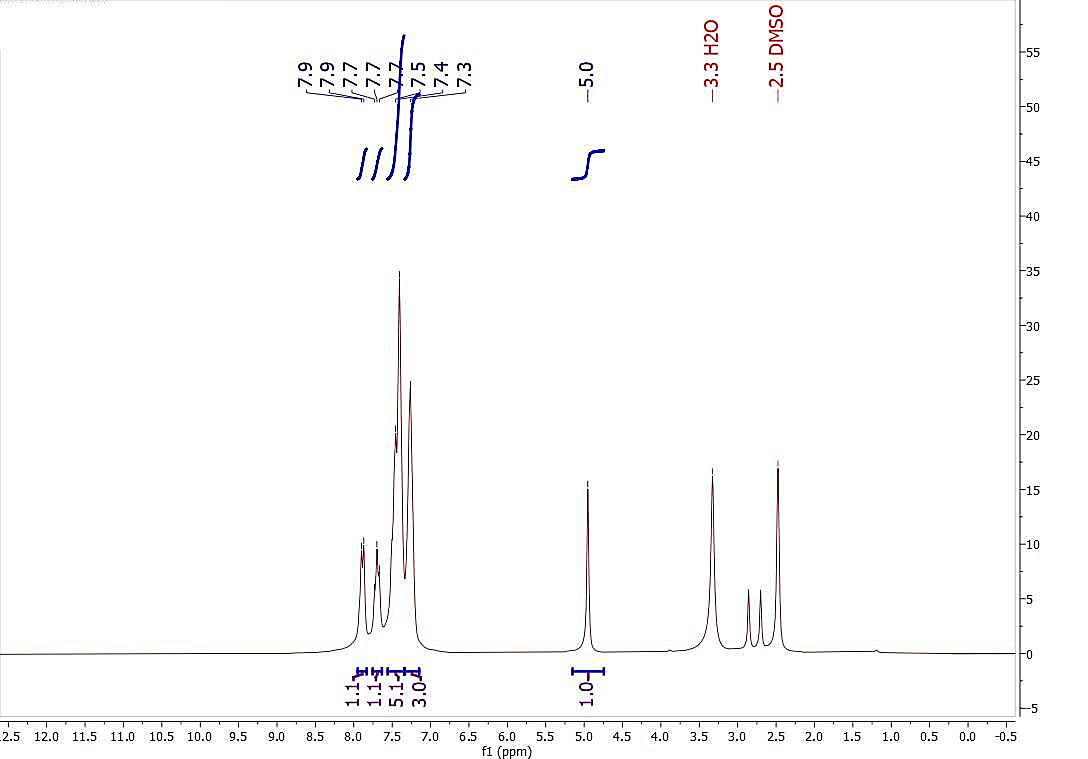


^1^H NMR Spectrum of *2-Amino-4-(2-chlorophenyl)-5-oxo-4H,5H-pyrano[3,2-c]chromene-3-carbonitrile* (**4d**)

^
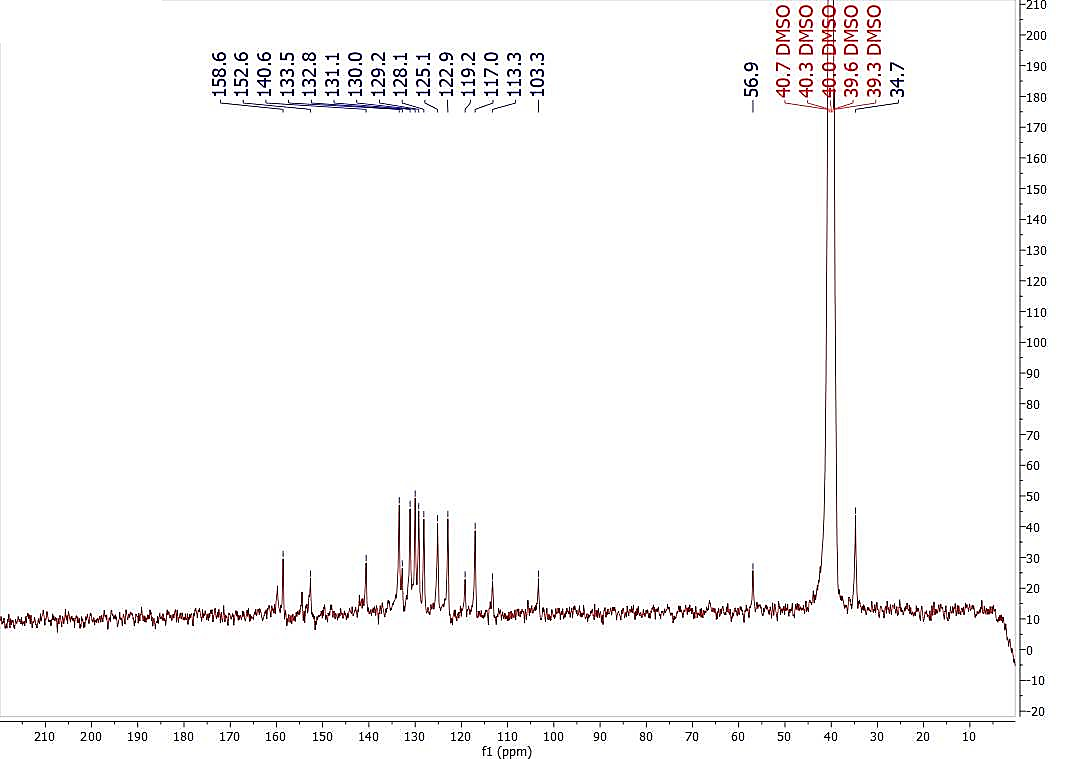
^

^13^C NMR Spectrum of *2-Amino-4-(2-chlorophenyl)-5-oxo-4H,5H-pyrano[3,2-c]chromene-3-carbonitrile* (**4d**)


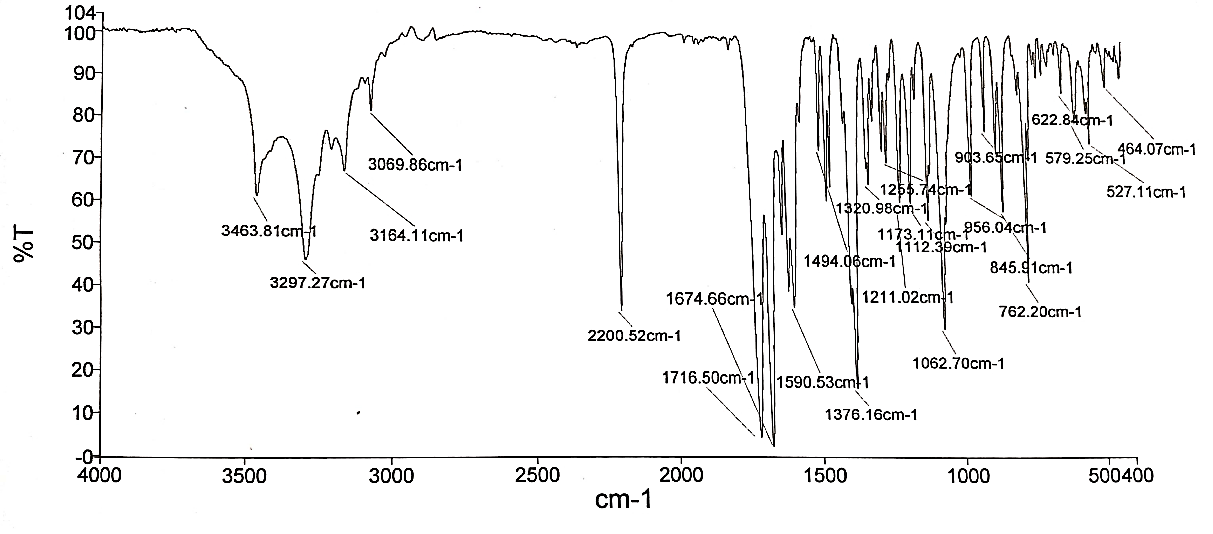


FT-IR Spectrum of *2-Amino-4-(2,4-dichlorophenyl)-5-oxo-4H,5H-pyrano[3,2-c]chromene-3-carbonitrile* (**4e**)


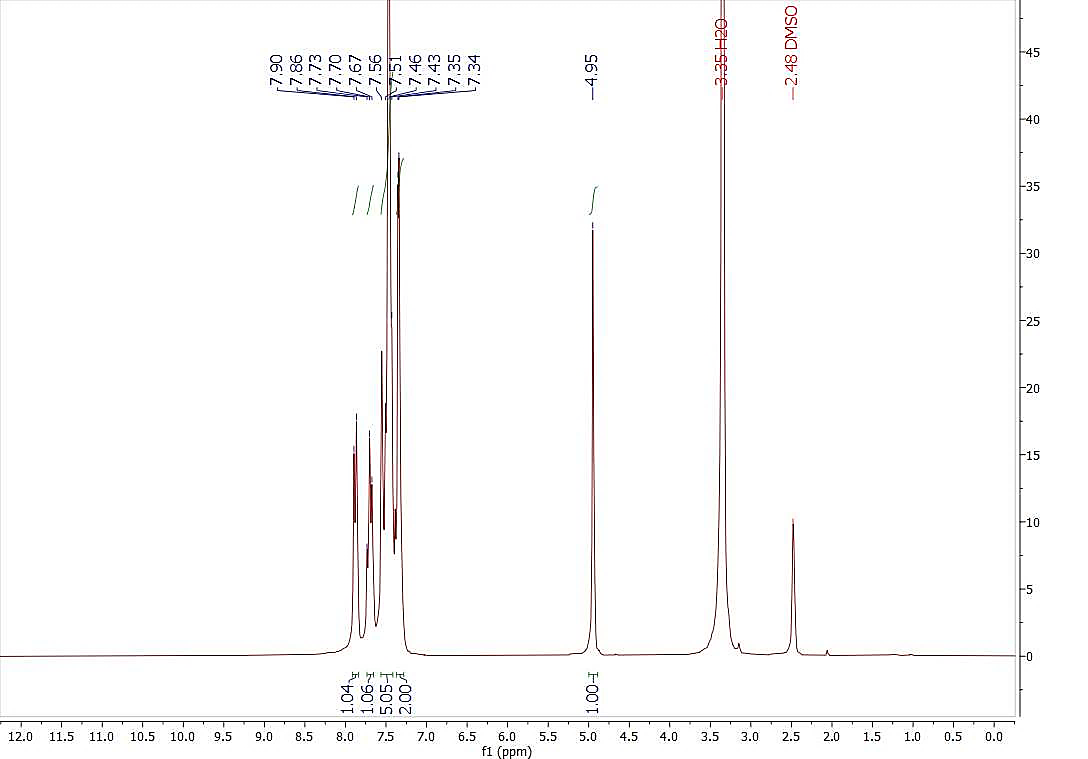


^1^H NMR Spectrum of *2-Amino-4-(2,4-dichlorophenyl)-5-oxo-4H,5H-pyrano[3,2-c]chromene-3-carbonitrile* (**4e**)


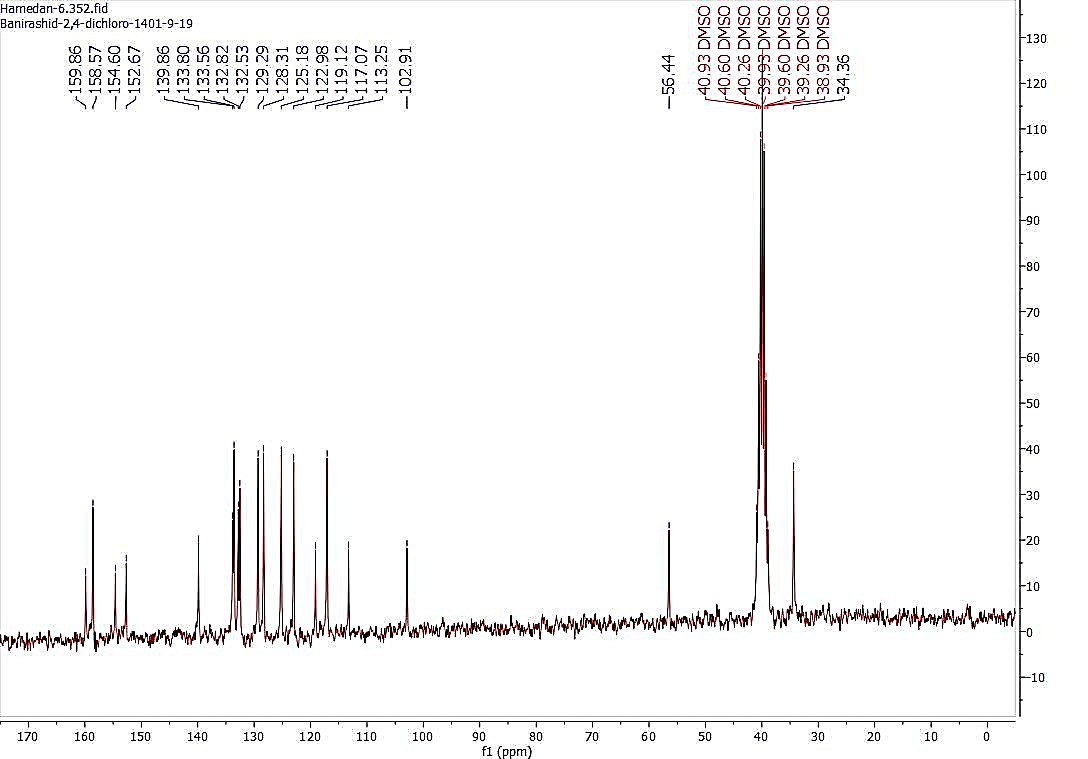


^13^C NMR Spectrum of *2-Amino-4-(2,4-dichlorophenyl)-5-oxo-4H,5H-pyrano[3,2-c]chromene-3-carbonitrile* (**4e**)


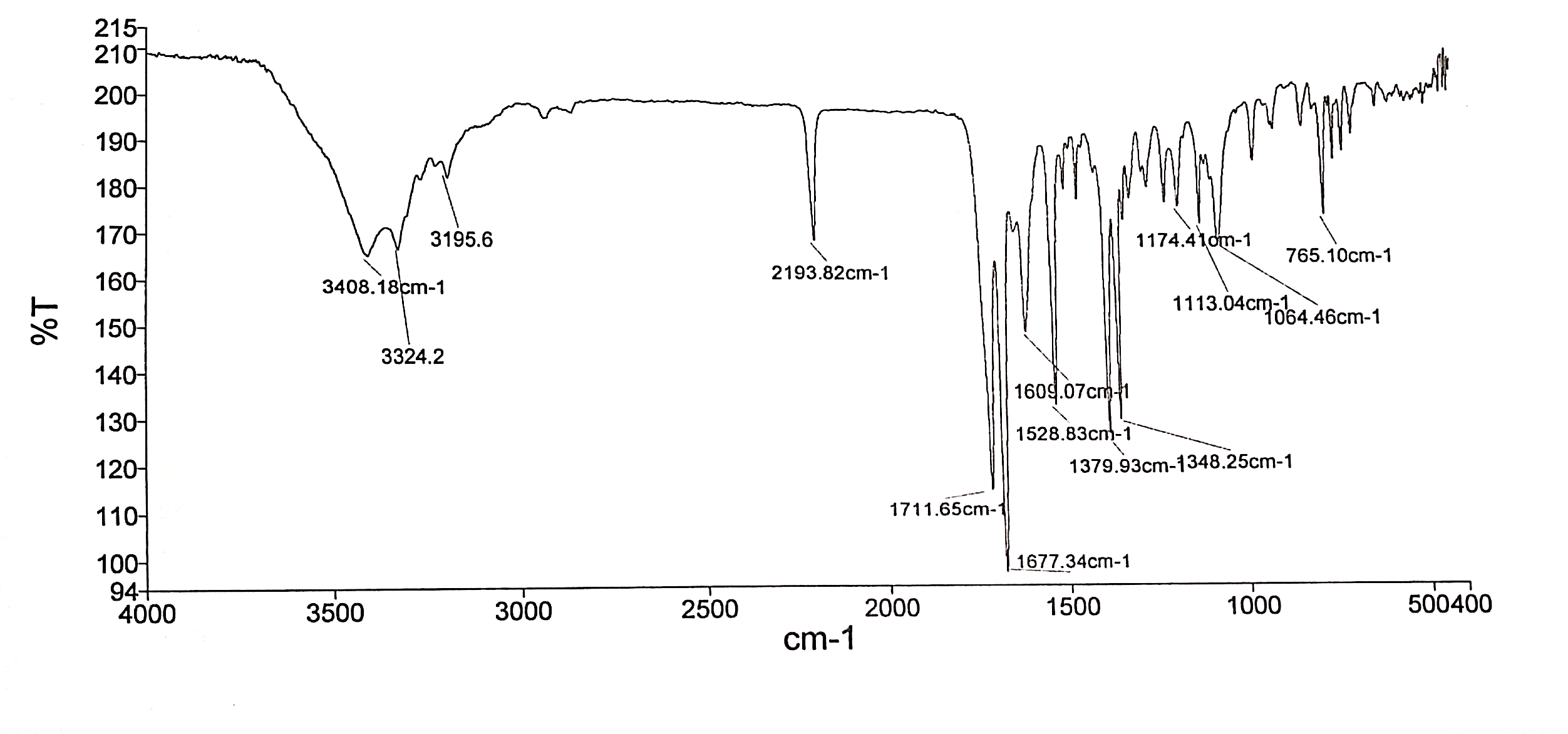


FT-IR Spectrum of *2-Amino-4-(3-nitrophenyl)-5-oxo-4H,5H-pyrano[3,2-c]chromene-3-carbonitrile* (**4f**)


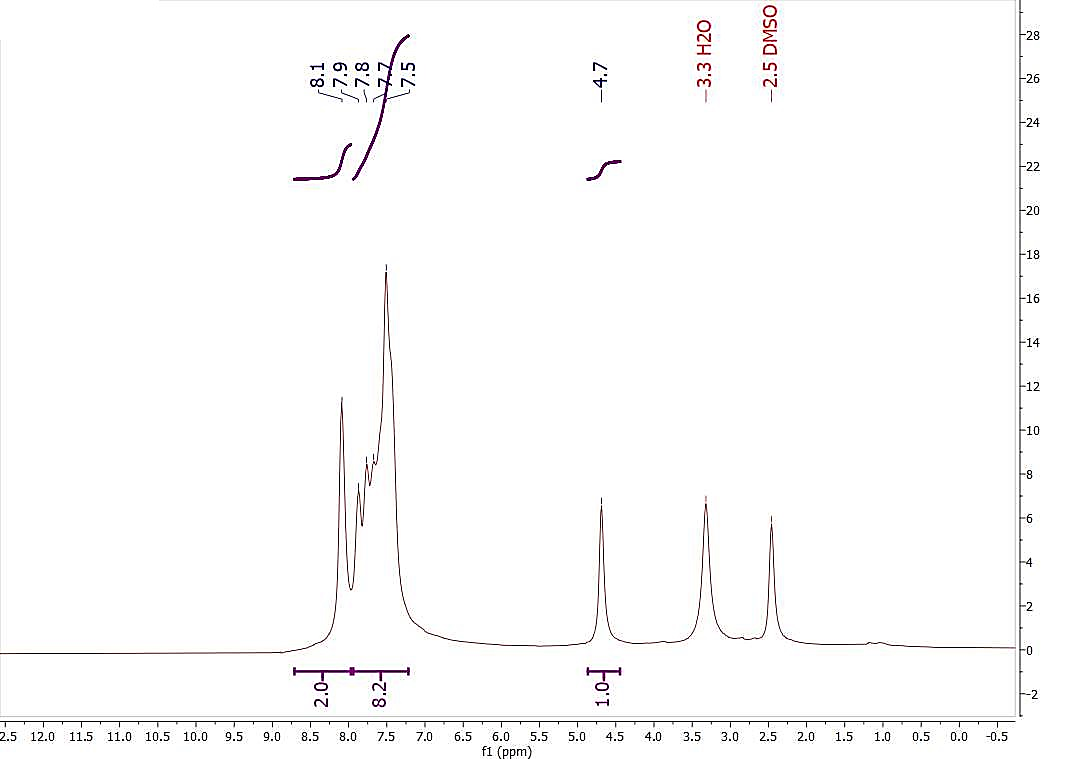


^1^H NMR Spectrum of *2-Amino-4-(3-nitrophenyl)-5-oxo-4H,5H-pyrano[3,2-c]chromene-3-carbonitrile* (**4f**)


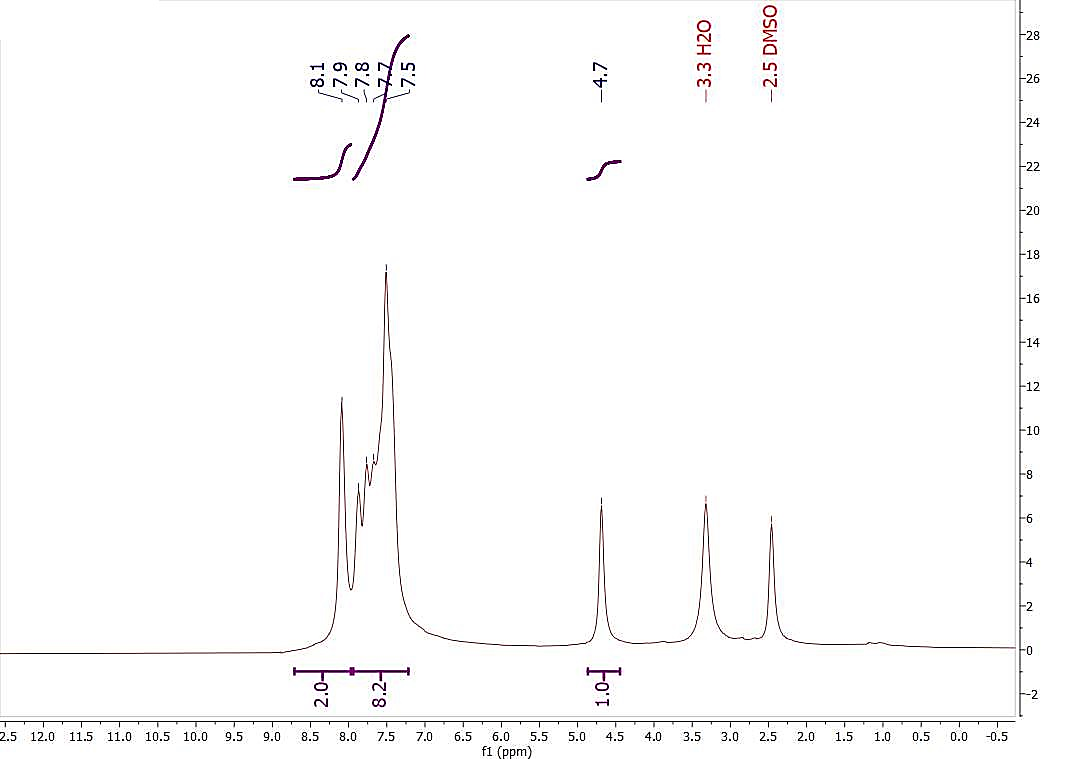


^13^C NMR Spectrum of *2-Amino-4-(3-nitrophenyl)-5-oxo-4H,5H-pyrano[3,2-c]chromene-3-carbonitrile* (**4f**)


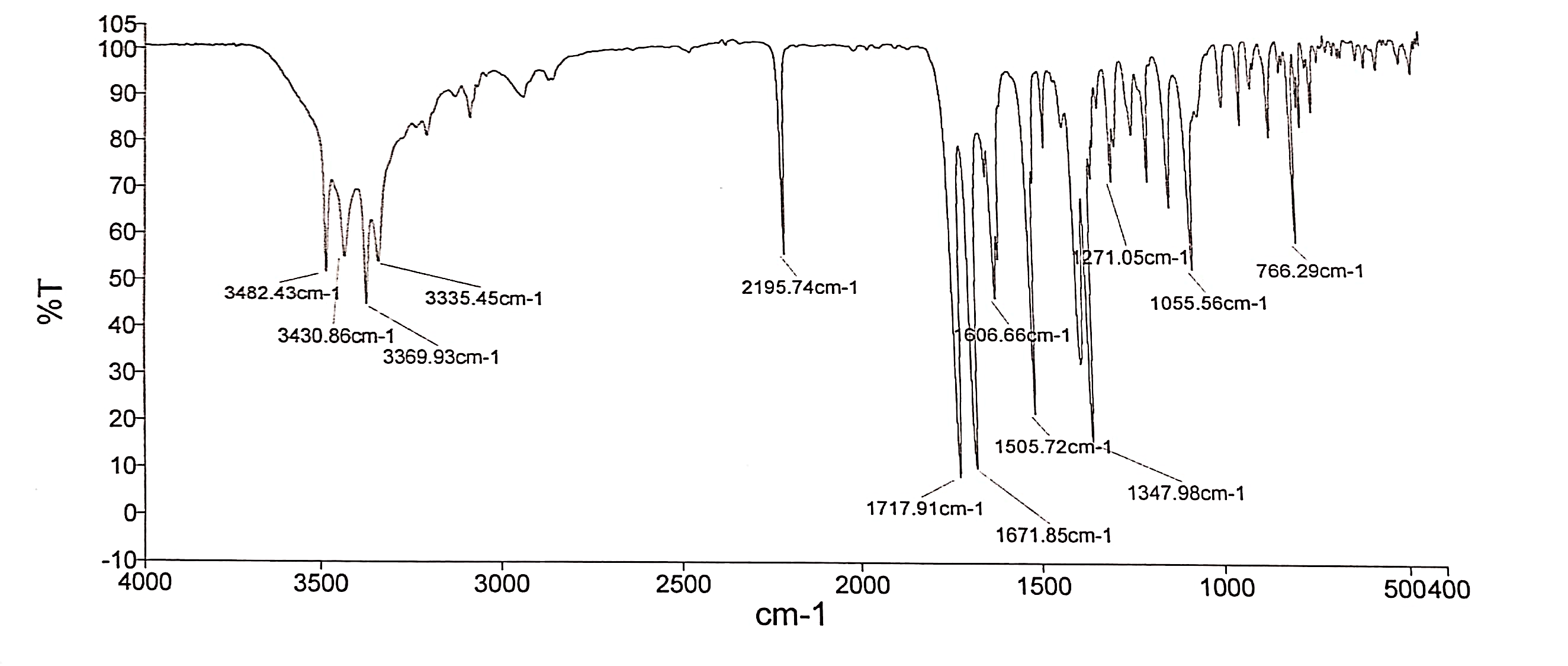


FT-IR Spectrum of *2-Amino-4-(4-nitrophenyl)-5-oxo-4H,5H-pyrano[3,2-c]chromene-3-carbonitrile* (**4g**)


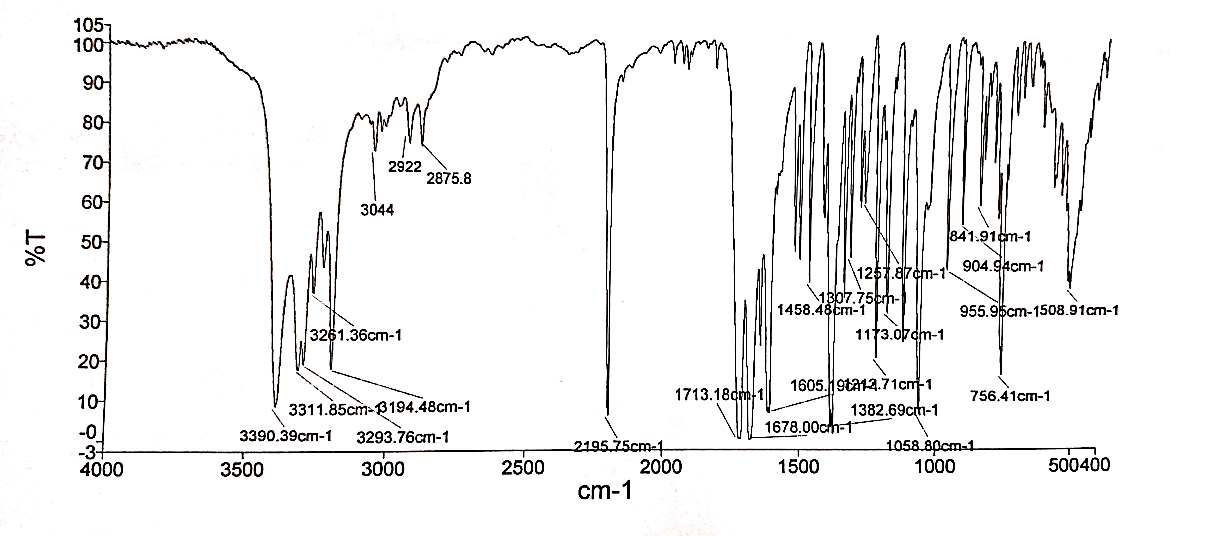


FT-IR Spectrum of *2-Amino-5-oxo-4-(p-tolyl)-4H,5H-pyrano[3,2-c]chromene-3-carbonitrile* (**4h**)


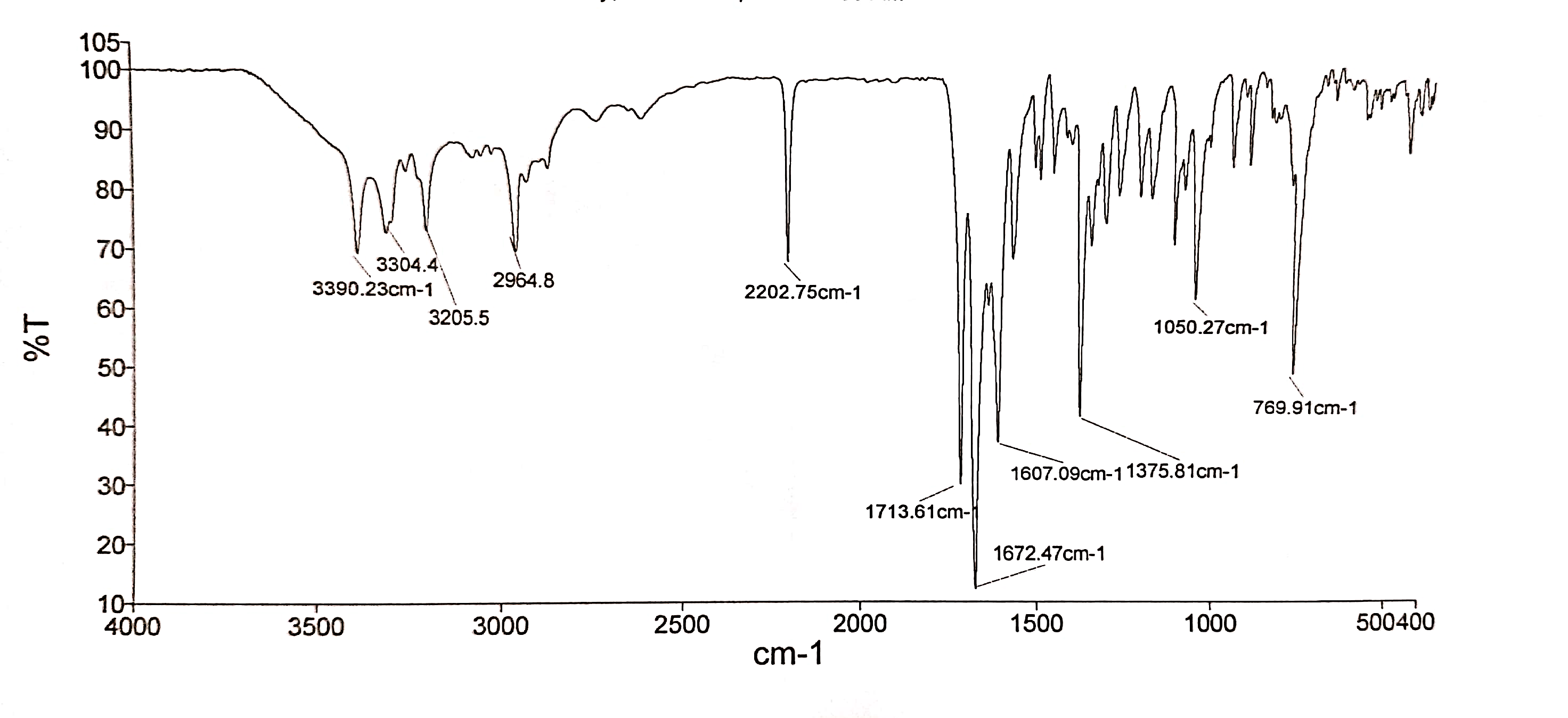


FT-IR Spectrum of *2-Amino-4-(4-isopropylphenyl)-5-oxo-4H,5H-pyrano[3,2-c]chromene-3-carbonitrile* (**4i**)


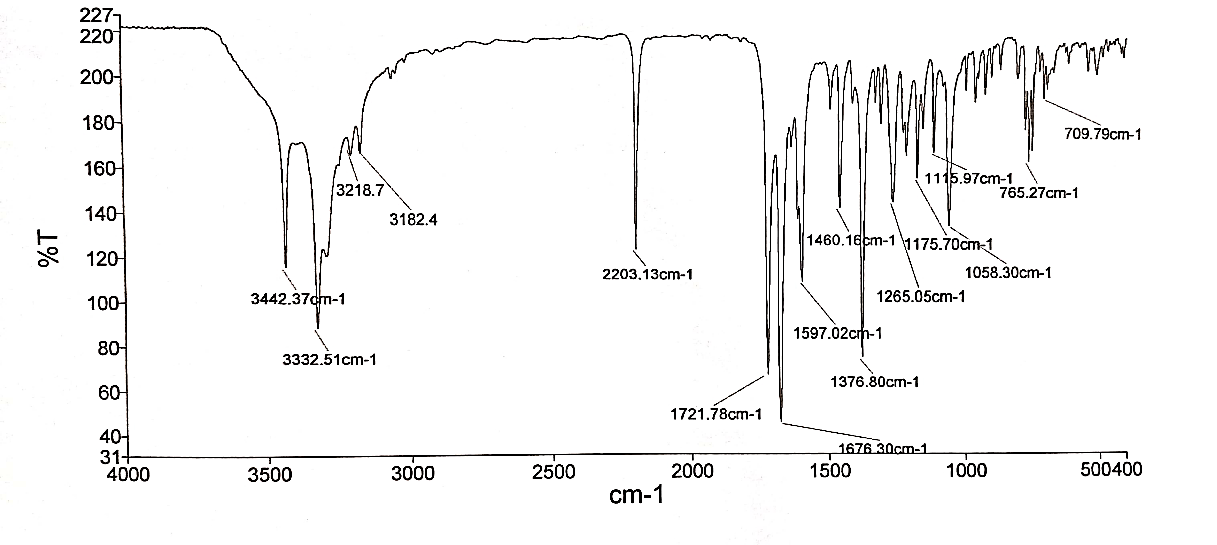


FT-IR Spectrum *2-Amino-4-(3-hydroxyphenyl)-5-oxo-4H,5H-pyrano[3,2-c]chromene-3-carbonitrile* (**4j**)


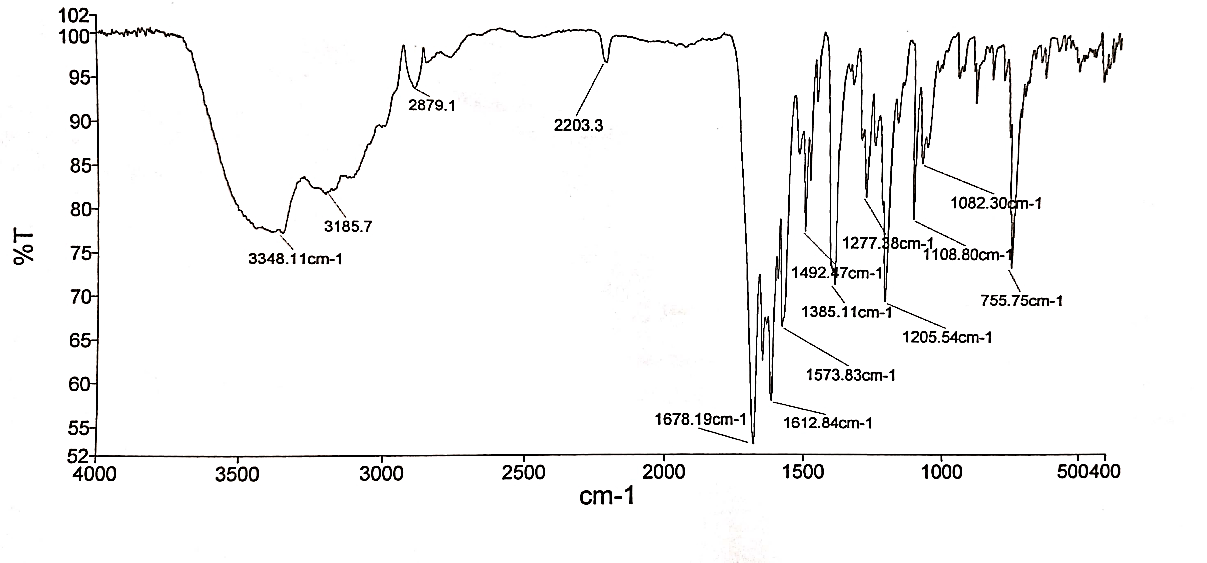


FT-IR Spectrum of *2-Amino-4-(2,3-dihydroxyphenyl)-5-oxo-4H,5H-pyrano[3,2-c]chromene-3-carbonitrile* (**4k**)


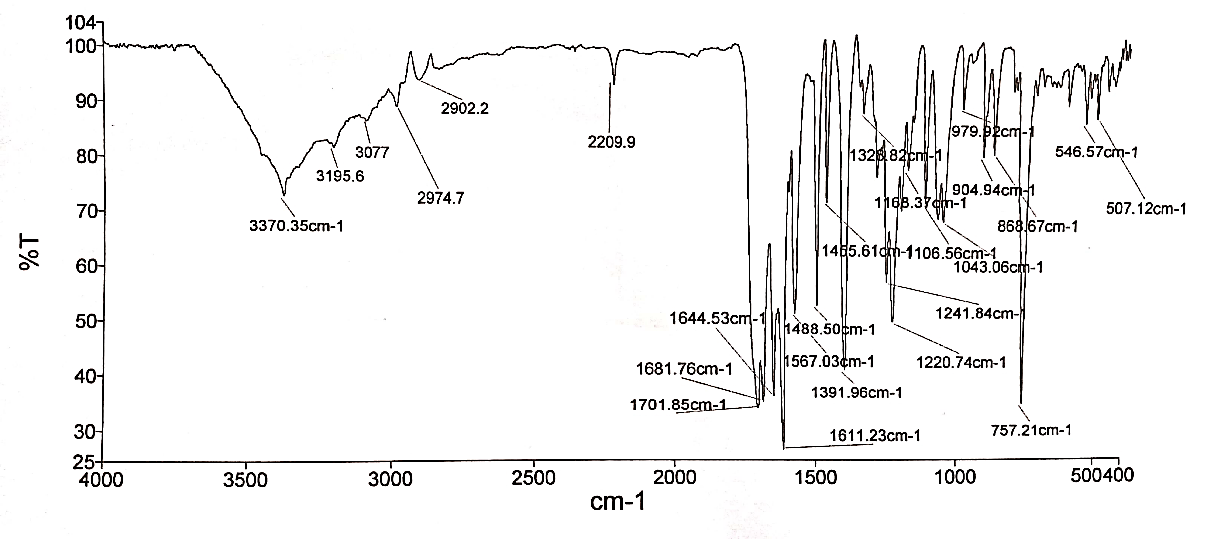


FT-IR Spectrum of *2-Amino-4-(2-hydroxyphenyl)-5-oxo-4H,5H-pyrano[3,2-c]chromene-3-carbonitrile* (**4l**)


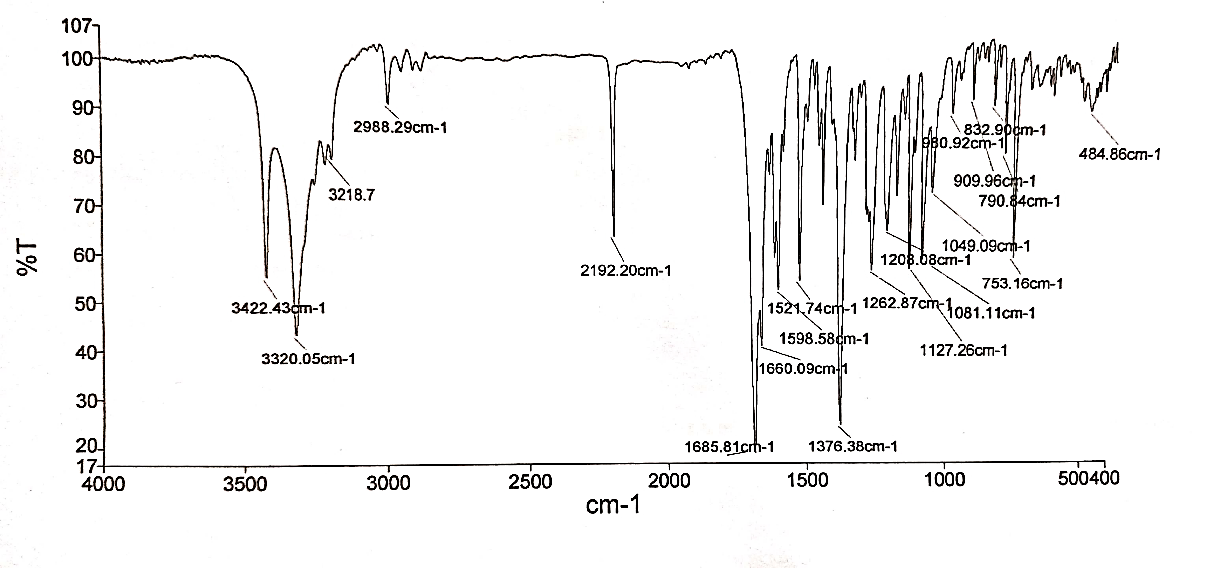


FT-IR Spectrum of *2-Amino-4-(3-ethoxy-4-hydroxyphenyl)-5-oxo-4H,5H-pyrano[3,2-c]chromene-3-carbonitrile* (**4m**)
